# Potent inhibition of human tyrosinase inhibitor by verproside from the whole plant of *Pseudolysimachion* *rotundum* var. *subintegrum*

**DOI:** 10.1080/14756366.2023.2252198

**Published:** 2023-08-30

**Authors:** Sunin Jung, So-Yeun Woo, Mi Hyeon Park, Doo-Young Kim, Su Ui Lee, Sei-Ryang Oh, Mun-Ock Kim, Jinhyuk Lee, Hyung Won Ryu

**Affiliations:** a Korea Research Institute of Bioscience and Biotechnology, Cheong-ju si, Republic of Korea; b Department of CBRN Medicine Research, center for Special Military Medicine, Armed Forces Medical Research Institute, Daejeon, South Korea; c Disease Target Structure Research Center, Korea Research Institute of Bioscience and Biotechnology (KRIBB), Daejeon, Republic of Korea; d Department of Bioinformatics, KRIBB School of Bioscience, University of Science and Technology (UST), Daejeon, Republic of Korea

**Keywords:** Human tyrosinase inhibitor, Verproside, *Pseudolysimachion rotundum* var. *subintegrum*

## Abstract

Affinity-based ultrafiltration–mass spectrometry coupled with ultraperformance liquid chromatography–quadrupole time-of-flight mass spectrometry was utilised for the structural identification of direct tyrosinase ligands from a crude *Pseudolysimachion rotundum* var. *subintegrum* extract. False positives were recognised by introducing time-dependent inhibition in the control for comparison. The *P. rotundum* extract contained nine main metabolites in the UPLC-QTOF-MS chromatogram. However, four metabolites were reduced after incubation with tyrosinase, indicating that these metabolites were bound to tyrosinase. The IC_50_ values of verproside (**1**) were 31.2 µM and 197.3 µM for mTyr and hTyr, respectively. Verproside showed 5.6-fold higher efficacy than that of its positive control (kojic acid in hTyr). The most potent tyrosinase inhibitor, verproside, features a 3,4-dihydroxybenzoic acid moiety on the iridoid glycoside and inhibits tyrosinase in a time-dependent and competitive manner. Among these three compounds, verproside is bound to the active site pocket with a docking energy of −6.9 kcal/mol and four hydrogen bonding interactions with HIS61 and HIS85.

## Graphical abstract

**Figure:**
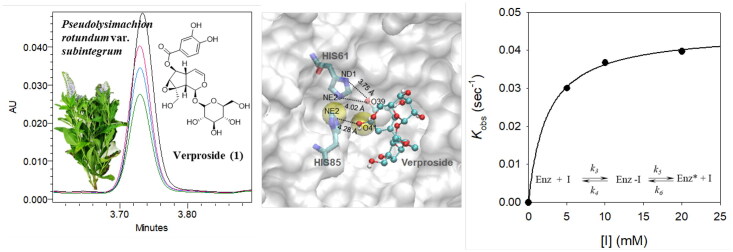

## Introduction

Hyperpigmentation of human skin offers the greatest protection against UV-induced damage^1–3^. Long-term hyperpigmentation leads to melanoma, melasma, freckles, ephelides, postinflammatory hyperpigmentation, and solar lentigines^2^^,^^3^. One way to ameliorate these negative symptoms is to create a direct inhibitor/enzyme interaction with the enzyme tyrosinase^3–6^. Tyrosinase (EC 1.14.18.1) is a multifunctional, glycosylated, copper-containing oxidase^7^ that catalyses the following reactions: the ortho-hydroxylation of monophenols (L-tyrosine) to *O*-diphenols (L-dopa), known as monophenolase (cresolase) activity, and the oxidation of *O*-diphenols (L-dopa) into reactive *O*-quinones (*O*-dopaquinone), known as diphenolase activity^7–9^. Thus, inhibitors of the tyrosinase mechanism might offer an effective strategy to control hyperpigmentation. Various strategies have been employed to inhibit tyrosinase activity, including the following: reducing dopaquinone (e.g. ascorbic acid) or using an o-dopaquinone scavenger (e.g. thio-containing compounds), alternative enzyme substrates (e.g. phenolic compounds), non-specific enzyme inactivators (e.g. acids or bases), or specific tyrosinase inactivators (e.g. mechanism-based inhibitors). Therefore, the development of more effective tyrosine inhibitors with direct inhibitor/enzyme interactions, especially targeted inhibitors, is an important task.

As a result, numerous medicinal compound discovery strategies have included methods to screen numerous natural products for the identification of novel biologically active components^10^^,^^11^. However, target compounds cannot be efficiently isolated because they decompose under the conditions of conventional repeated column chromatography isolation, they are present at an extremely low concentration, and/or their actions may occur due to the complex synergistic effect of multicomponent multitargets (MCMTs)^12–14^. Therefore, an affinity ultrafiltration liquid chromatography–mass spectrometry (LC–MS) method was recently developed to screen enzyme inhibitors from plant extracts and identify lead compounds^15–18^. In principle, these enzyme inhibitors must form a ligand–receptor complex that is retained from the extract so that the active compounds can be easily identified by LC–MS^19^^,^^20^. This approach to natural product screening is an effective strategy for obtaining more active compounds.

Iridoids are widely biosynthesized in plants and possess iridodials joined by glycoside and non-glycoside systems, and these iridoid glycosides can be further subdivided into carbocyclic iridoids and secoiridoids. Iridoid glycosides have received much attention due to the wide variety of pharmacological activities reported for these compounds, including anti-inflammation, anti-diabetic, anti-cardiovascular, and anti-cancer properties. Although iridoids has been shown to induce reduce the growth of mushroom tyrosinase^21^^,^^22^, the anti-tyrosinase effects of iridoid glycosides in enzyme kinetics and human tyrosinase have not been widely studied.

In this research, we investigated the possibility of using competitive binding metabolites with affinity ultrafiltration and quadrupole time-of-flight mass spectrometry (QTOF)-MS analysis to identify target tyrosinase inhibitors from the whole plant of *Pseudolysimachion rotundum* var. *subintegrum*. A binding affinity test with ultraperformance liquid chromatography QTOF (UPLC-QTOF-MS) identified nine iridoids that appeared to interact strongly with tyrosinase. Among the nine isolated compounds examined, verproside (**1**) was found to effectively inhibit tyrosinase activity. Notably, affinity ultrafiltration, enzyme kinetics, and docking simulations of potent active compound **1** showed that the compound inhibits tyrosinase through direct competitive binding and time-dependent inhibition.

## Materials and methods

### General experimental procedures

High-speed counter-current chromatography (HSCCC) was carried out with a TBE-1000A high-speed countercurrent chromatography system (Shanghai Tauto Biotech Co. Ltd., Shanghai, China) with three serially connected multilayer coil separation columns (tubing = 1.6 mm; total volume = 1000 ml) and an 80 ml sample loop. The system was equipped with an LPLC pump (TBP5002, Shanghai Tauto Biotech Co. Ltd., Shanghai, China), a Thermo Finnigan SSI 500 UV detector (Thermo Electron Co., San Jose, CA), and Autochro-WIN software (version 3.0, Younglin-Tech, Seoul, Korea). ^1^H and ^13^C NMR spectra were recorded on a Bruker AM500 instrument (Billerica, Massachusetts, USA). Profiling and tentative identification of the compounds were achieved with ultrahigh-performance liquid chromatography coupled with electrospray ionisation and quadrupole time-of-flight mass spectrometry (UPLC-ESI-QTOF-MS; ACQUITY UPLC™ system coupled to a Micro mass QT Premier™, Waters, Milford, MA, USA) with a reversed-phase column (ACQUITY BEH C18 1.7 μm, 2.1 × 100 mm, Waters). Purified water was obtained from a Milli-Q Academic system (Merck Millipore, Burlington, MA, USA). The reagents required for UPLC-ESI-QTOF-MS analysis were acetonitrile (ACN) (Merck Millipore), formic acid, and leucine enkephalin (Sigma–Aldrich, St. Louis, MO, USA). Solvents for NMR were purchased from Cambridge Isotope Lab Inc. (Andover, MA, USA).

### Plant material

The whole plant of *P. rotundum* var. *subintegrum* was collected at Eumsung Chungbuk, Korea, in September 2011 and identified by Min-Ha Kim from the National Institute of Biological Resources. For recollection, seeds of *Pseudolysimachion rotundum* var. *subintegrum* were cultivated in a well-controlled glass greenhouse at the KRIBB, Cheong-ju, Korea in 2020. A voucher specimen (KRIB 0020697) was deposited at the Plant Extract Bank of KRIBB in Daejeon, Korea. Each of the collected plants were divided into two parts, the aerial parts and roots, and chopped and extracted at room temperature (as described below). The aerial parts were packaged and transported to a laboratory (over a maximum of 2 h) using refrigerating equipment and were stored at − 20 °C in Ziplock bags (Ziploc, S. C. Johnson & Son, Inc., Racine, WI, USA) for a maximum of 20 wk. The *P. rotundum* var. *subintegrum* aerial parts were immediately freeze-dried (CleanVac 8 HanilScience Medical, Daejeon, Korea), pulverised (Model 0004180000, tubemill control, IKA, Staufen, Germany) and stored in the dark at −20 °C for a maximum of 20 wk.

### Affinity-based ultrafiltration procedures

Affinity-based ultrafiltration was performed as previously described by Zuo et al. and Wang et al.^15^^,^^19^^,^^23^. First, 2487 μL of 0.25 M phosphate buffer (pH 6.8) with 10% glycerol was added to 13 μL of mushroom tyrosinase (144 U in the same phosphate buffer solution) and 100 μL of 1000 mg/mL extract (the test solution was consistently 3.3%). The final concentration of the extract was adjusted to 1000 ppm. The mixture was incubated for 15 or 50 min at 37 °C on a plate shaker. Then, the sample was centrifuged at 13,000 rpm for 20 min, and the supernatant was collected for analysis. The supernatant solution was filtered through a membrane (PTFE 0.2 μm, hydrophobic, Advantec, Japan), and 2 μL of each solution was injected for UPLC-PDA-QTOF-MS analysis.

### UPLC-PDA-ESI-QTOF-MS chromatographic conditions

The compounds from the *P. rotundum var. subintegrum* extract were analysed by UPLC-PDA-ESI-QTOF-MS. The chromatographic gradient program included two mobile phase solutions (A: water with 0.1% formic acid; B: ACN with 0.1% formic acid) was as follows: 0–1 min, 10% B; 1–10.5 min, 10–23% B; 10.5–12.0 min, 23–98% B; 12.0–13.3 min, 98% B; 13.3–13.4 min, 98–10% B and maintained at 10% B. Analysis was performed at a flow rate of 0.4 ml/min. MS was performed in negative ion mode ([M − H]^−^). N_2_ was used as the desolvation gas, the desolvation temperature was set to 350 °C at a flow rate of 400 L/h with a source temperature of 100 °C. The capillary and cone voltages were set to 2300 and 50 V, respectively. The data for each sample were collected with a scan time of 0.25 s and a 0.01 s interscan delay. Exact MS, MS/MS, and elemental analysis were confirmed using MassLynx software incorporated into the Waters instrument.

### Extraction and isolation of iridoids

The dried whole plant of *P. rotundum* var. *subintegrum* (2 kg) was extracted with MeOH (10 L × 3) at room temperature, and the solvent was concentrated using an evaporator under reduced pressure at 40 °C to obtain the methanolic extract (198.7 g). A portion of the methanolic extract (30 g) was subjected to C18 column chromatography using MeOH-H_2_O (3:97, 25:75, 65:35, 100:0 *v*/*v*) to give four fractions (PL-1 to PL-4). Fraction PL3 (5 g) was further fractionated by medium-pressure liquid chromatography (MPLC) with an RP C-18 column (5 cm × 49 cm, 40 C_18_-prep) eluted with MeOH-H_2_O (20:80, 30:70, 40:60 *v*/*v, step gradient*) to afford five fractions (PL3m–1 to PL3m–5). Fraction PL3m–1 (1.2 g) was subjected to preparative HPLC using MeOH-H_2_O (1:2, *v*/*v*) to give compound **1** (500 mg). Fraction PL3m–2 (800 mg) was purified by Sephadex LH-20 (2.5 × 170 cm) column chromatography eluted with 90% MeOH to give four subfractions, PL3m–2–1 to PL3m–2–4. Subfraction PL3m–2–1 (52.6 mg) was separated by preparative HPLC using an Atlantis® prep T_3_ OBD^TM^ column (5 μm particle size, 19 × 250 mm; mobile phase of 18% ACN in H_2_O; flow rate of 16 ml/min; UV detection at 263 nm] to obtain compounds **2** (15.1 mg) and **3** (12.5 mg). Fraction PL3m–4 (18.3 mg) was isolated by preparative HPLC [Atlantis^®^ prep T_3_ OBD^TM^ column (5 μm particle size, 19 × 250 mm); mobile phase 18% of ACN in H_2_O; flow rate 16 ml/min; UV detection at 263 nm] to give compound **4** (5.4 mg). Compounds **5** (19.7 mg), **6** (11.3 mg), and **7** (28.7 mg) were obtained from the purification of a portion of fraction PL3m–4 (2.8 g) by HSCCC [*n*-BuOH/H_2_O = 1:1 *v*/*v*, upper phase for the stationary phase, lower phase for the mobile phase; loading sample: 100 mg; flow rate: 5.5 ml/min; 600 rpm; forward; UV: 263 nm]. Fraction PL3m–5 (50.6 mg) was further separated with an RP-C18 MPLC column eluting with a gradient of MeOH-H_2_O (4:6, 5:5, 6:4, 7:3, 10:0) to afford compounds **8** (2.1 mg) and **9** (1.9 mg).

### Tyrosinase kinetic assay

The enzymatic activities of tyrosinase were tested *in vitro* with the substrates and inhibitors known to interact with mushroom and mammalian tyrosinases (http://www.brenda-enzymes.info). The Michaelis-Menten constant (*K*_m_) of proteins was calculated from Lineweaver-Burk plots for 1/*V*_0_ versus 1/[substrate]. Mushroom tyrosinase (mTyr, EC 1.14.18.1, Sigma Chemical Co., T3824-50KU) was used as described previously^9^ with some modifications with either 3,4-Dihydroxy-L-phenylalanine (L-DOPA, D9628-5G) or L-tyrosine (T3754-50G) as the substrate. In the spectrophotometric experiments, the initial velocity (*v_i_*) of enzymatic activity was monitored by observing dopachrome formation at 475 nm with a UV–vis spectrophotometer (SpectraMax M4, Molecular Devices, Sunnyvale, CA, USA) at 37 °C. The reaction was performed in a 96-well plate. All samples were first dissolved in dimethyl sulfoxide (DMSO) at a concentration of 2 mM. First, 15 μL of a 2.7 mM L-tyrosine (*K*_m_ = 180 μM) or 10 μL of 7 mM L-DOPA (*K*_m_ = 350 μM) aqueous solution was mixed with 0.25 M phosphate buffer (pH 6.8). Then, 10 μL of the sample solution and 2 μL of the same phosphate buffer solution containing mushroom tyrosinase (144 U) were added to the mixture in sequence (unit definition: one unit will cause an increase in A_280_ of 0.001 per minute at pH 6.5 and 25 °C in a 3 ml reaction system containing L-tyrosine). The activity of recombinant human tyrosinas (hTyr, His-tag, Enzo^®^ Life Sciences, NY, USA, Cat. No. BML-SE535-0100) was determined using L-DOPA as a substrate, and the procedure involved a previously described absorption assay^24^. Each well contained a mixture 20 μL of 7.0 mM L-DOPA (*K*_m_ = 630 μM) and 2 μL of 1 mg/mL protein concentrate in 50 mM Tris-HCl, pH 8.0, 1 M NaCl, 0.05% empigen BB, and 30% glycerol (Purity: ≥85%, SDS-PAGE). The reaction was incubated for 10 min at 37 °C and was monitored at 475 nm for dopachrome formation on a SpectraMax M4 multimode detection platform (Molecular Devices). Each assay was conducted as three separate replicates. The inhibitory concentration leading to 50% activity loss (IC_50_) was determined by fitting the experimental data to the logistic curve according to the following equation^25^:

(1) $$\text{Activity }\left(\%\right)\;=\text{ 1}00[1/(1\;+\;([I]/{\text{IC}}_{50}))]$$

### Affinity-based time-dependent assay

Time-dependent assays were carried out using 144 U of mushroom tyrosinase and L-tyrosine as the substrate in 0.25 M phosphate buffer (pH 6.8) at 30 °C. To determine the kinetic parameters associated with the time-dependent inhibition of tyrosinase, progress curves were constructed from the chromatograms after preincubation for 5, 15, 20, 25, and 30 min with a fixed inhibitor concentration. The data were analysed using a nonlinear regression program [SigmaPlot (SPCC Inc., Chicago, IL)] to give the individual parameters for each curve; *v*_i_ (initial velocity), *v*_s_ (steady–state velocity), *k*_obs_ (apparent first–order rate constant for the transition from *v*_i_ to *v*_s_), *A* (intensity), *A*_0_ (included to correct any possible deviation in the baseline), and *K*_i_^app^ (apparent *K*_i_) according to Equations. 2–4^26–28^:

(2) $$A=v_{s}\text{t }+\;\left(v_{i}–v_{s}\right)\left[1\;–\text{ exp }\left(–k_{\text{obs}}t\right)\right]/k_{\text{obs}}$$

(3) $$v/v_{0}=\text{ exp}\left(-k_{\text{obs}}t\right)$$

(4) $$k_{\text{obs}}=k_{6}+\;[(k_{5}\;\times \;[I])/(K_{i}{}^{\text{app}}\;+\;[I])]$$

### Tyrosinase docking and MD simulations

In silico docking simulations of nine compounds with tyrosinase were performed using AutoDock Vina^29^. The three-dimensional structure of tyrosinase was obtained from the Protein Data Bank (PDB) with ID, 2Y9X. The protein structure originated from Agaricus bisporus. The human origin tyrosinase structure has not been revealed experimentally and was predicted using alpha fold program (https://www.uniprot.org/uniprotkb/P14679/). Because the mushroom tyrosinase (mTyr) is the experimental structure, the docking simulations were performed mainly in the tyrosinase. The human tyrosinase (hTyr) was used for comparison. Because tyrosinase is a metalloenzyme and two Copper ions play important roles in its chemical reactions, these ions were included in the docking simulations. Nine compounds were constructed, and their structures were minimised using the Marvin program (ChemAxon; http://www.chemaxon.com). For docking simulations, the tyrosinase pockets were searched using the Pck pocket detection program (http://schwarz.benjamin.free.fr/Work/Pck/home.htm). Twelve pockets with volumes greater than 100.0 Å^3^ were found. Docking simulations focused on these pockets, which consisted of 100 pocket residues. Each compound was placed in the 100 pocket residues and the simulations were run ten times with different random seeds for a total of 1000 simulations. A box with a length of 15 Å was used to prevent the compounds from drifting from the centre of the pocket residue. With the obtained 1000 docking poses, clustering based on the centre of mass was performed using CHARMM (Chemistry at HARvard Macromolecular Mechanics) to group these conformations^30^. The compounds in each group were ranked by lowest energy, the number of compounds occupying the pocket, and average energy. The molecular structures were drawn with the visual molecular dynamics (VMD) visualiser program (https://www.ks.uiuc.edu/Research/vmd/). The hydrogen bonding interactions between each compound and tyrosinase were calculated based on geometric analysis. First, the hydrogen bond acceptor and donor atoms from both tyrosinase and the compounds were listed. All pairs of acceptor and donor atoms were then arranged to measure the geometric distances and angles. The hydrogen bonding criteria were less than 4 Å between the acceptor and donor atoms and an angle of over 120 degrees for the three atoms (donor, hydrogen, and acceptor).

Using the potent docking inhibitor, we performed molecular dynamics (MD) simulations with TIP3P explicit water solvation. The input scripts for MD were prepared using the Input Generator-Solution Builder of CHARMM-GUI (https://charmm-gui.org/). The protein-inhibitor model was constructed with TIP3 water molecules and KCl ions to obtain an electrically neutral system. MD simulation was carried our for 100 nanoseconds (ns) with the CHARMM36m force fields using GROMACS (v5.1.2) program. Trajectory data was recorded every 100 picoseconds (ps), resulting in a total of 1,000 frames. The MD integrator utilised utilized the leap-frog algorithm for integrating Newton’s equations of motion. All bonds were constrained using the linear constraint solver (LINCS) algorithm. Additionally, the MD simulation was conducted at a temperature of 303.15 K and pressure of 1 atm. Short minimisation and equilibrium dynamics were performed before the production simulation. The Nose-Hoover algorithm was applied to set the temperature boundary conditions, and the Parrinello-Rahman pressure coupling method was used for pressure boundary conditions. To assess the stability between tyrosinase and the inhibitor ligand, we calculated the root mean square fluctuation (RMSF) for C-alpha atoms of the protein, plotted the root mean square deviation (RMSD) versus time for the protein backbone structure, and determined the radius of gyration (Rg). Each calculation was executed using the built-in functions of GROMACS, specifically gmx rmsf, gmx rms, gmx gyrate, and visualised through python matplotlib. To build the distance profile between tyrosinase and the inhibitor, we utilised the dist module and visualised the results.

## Results and discussion

### Identification of tyrosinase inhibitors using affinity-based ultrafiltration–mass spectrometry combined with competitive binding experiments

**Figure 1.**
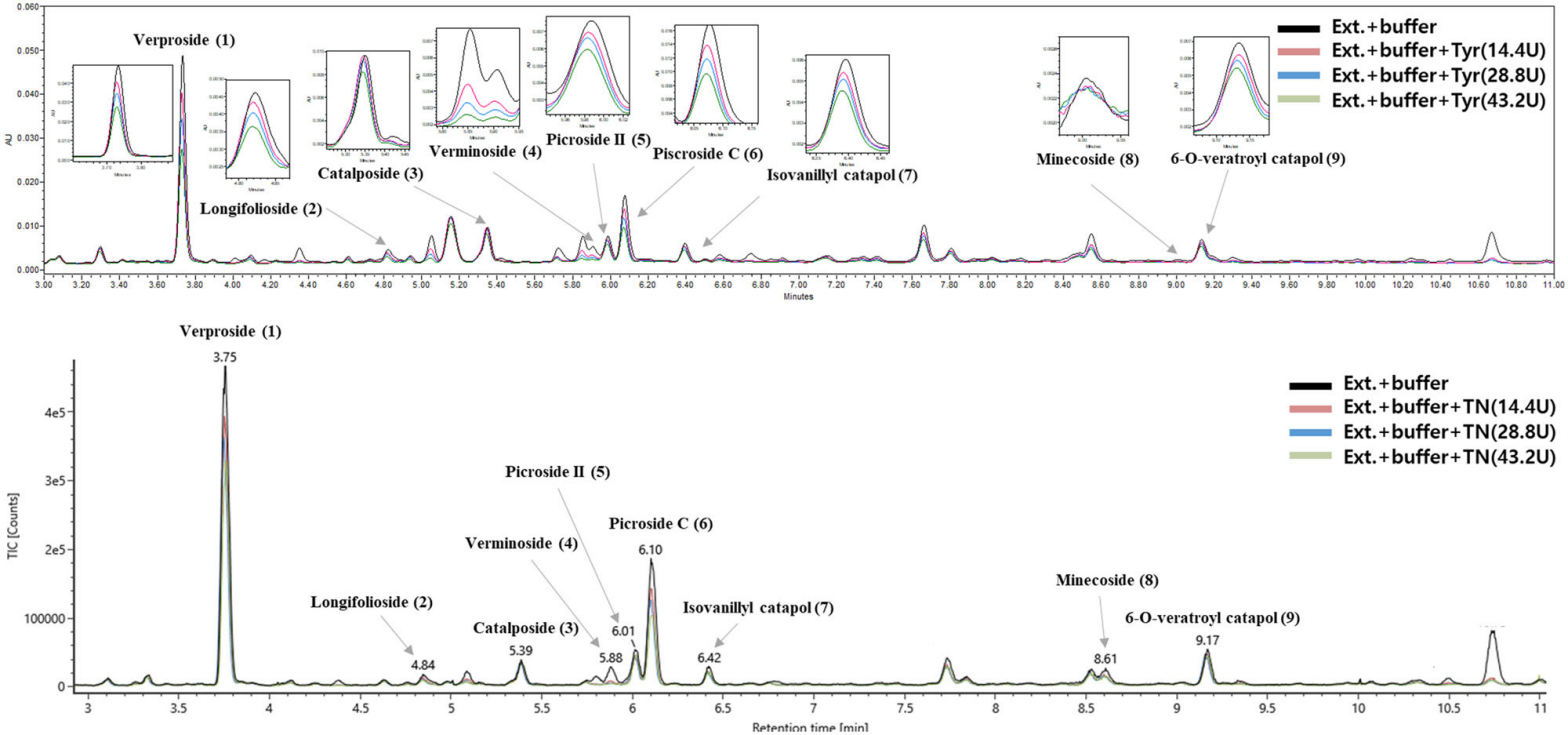
(A) UPLC-PDA and (B) UPLC-QTOF-MS data of the *Pseudolysimachion rotundum* var. *subintegrum* extract after the binding experiment. Black: not exposed to enzyme; red, blue, and green; after incubation with tyrosinase for 15 min. Peaks numbered **1 **−** 9** represent the compounds isolated from *P. rotundum* var. *subintegrum* in our study.

In most cases, enzyme binding affinity can be used to identify inhibitors of target proteins^31^. For this inhibition to occur, a reversible complex (Enz-I) formation step between the target protein (Enz) and inhibitor (I) and an irreversible dissociation step of Enz-I must occur. In addition, according to previous studies, most reported time-dependent inhibitors bind competitively^32^. Therefore, as a strategy to find a target inhibitor, we first optimised the experimental conditions, including enzyme concentration and preincubation time, to reduce background noise for distinguishing false hits from competitive inhibitors. As shown in Figure 1, a screening system based on UPLC-QTOF-MS was constructed to screen for tyrosinase-specific inhibitors from plants. The affinity-based ultrafiltration incorporated an 14.4, 28.8, and 43.2 U enzyme concentration and 1000 ppm extract and preincubation for 15 min followed by centrifugation and analysis using UPLC-PDA-QTOF-MS. By comparing the LC–MS chromatograms from the performed experiments, such as those in the presence or absence of enzyme and those with incubation times, the metabolites that were dramatically reduced when the enzyme was present under the optimal conditions were determined to be correlated with binding. The relative metabolite intensities of six compounds (**1**, **2**, **4**, and **6 **−** 8**) from the *P. rotundum* var. *subintegrum* extract were significantly reduced upon treatment with mTyr (Table 1). This result indicates that six compounds (**1**, **2**, **4**, and **6 **−** 8**) corresponding to metabolites have significant binding interactions with the target enzyme.

**Table 1.** UPLC chromatographic, UV–Vis, and mass spectral data of the compounds identified from *P. rotundum* var. subintegrum.

Peak	*t_R_* (min)	λ_max_ (nm)	Detected ion [M − H]^−^	Calculated ion [M − H]^−^	ppm	Molecular Formula	Identification	Control (area)	after tyrosinase (area)
1	3.75	296, 263, 220	497.1292	497.1295	−0.6	C_22_H_26_O_13_	Verproside	112306	61907
2	4.84	296, 263, 220	533.1068	533.1062	1.1	C_22_H_27_ClO_13_	Longifolioside A	10677	3852
3	5.39	258	481.1327	481.1346	−3.9	C_22_H_26_O_12_	Catalposide	26121	21250
4	5.88	328, 218	523.1436	523.1452	−3.1	C_24_H_28_O_13_	Verminoside	16481	2886
5	6.01	294, 263, 220	511.1451	511.1452	−0.2	C_23_H_28_O_13_	Picroside II	17798	12194
6	6.10	296, 263, 220	533.1064	533.1062	0.4	C_22_H_27_ClO_13_	Piscroside C	44999	22171
7	6.42	296, 263, 220	511.1447	511.1452	−0.1	C_23_H_28_O_13_	Isovanillyl catapol	12844	8093
8	9.04	327, 220	537.1647	537.1608	7.3	C_25_H_30_O_13_	Minecoside	976	465
9	9.17	328, 220	525.1627	525.1613	2.5	C_24_H_30_O_13_	6-*O*-Veratroyl catalpol	15756	11932

### UPLC-ESI-QTOF-MS analysis of the extract

The methanolic extract of *P. rotundum* var. *subintegrum* was analysed by UPLC-ESI-QTOF-MS. *P. rotundum* var. *subintegrum* belongs to the family Scrophulariaceae and is rich in iridoid glycosides^33^. More sensitive MS detection of iridoid glycosides occurs in negative ionisation mode than in positive ionisation mode^34^^,^^35^. Therefore, we identified all isolated compounds (**1 **−** 9**) in negative mode, and all data were detected from the deprotonated molecule [M − H]^−^ in the MS/MS spectra (Figure 1B and S3). The UV spectrum showed absorption peaks at 220, 263 and 296 nm, indicating the presence of a conjugated enol–ether system. High-resolution–electrospray ionisation–mass spectrometry (HRESIMS) analysis of the molecular ion cluster [M − H]^−^ (*m/z* 497.1292) of verproside (**1**), as the major compound in *P. rotundum* var. *subintegrum*, established the molecular formula as C_20_H_20_O_5_. The MS fragment detected at *m/z* 335.0784 [M − H − 162] ^−^ corresponded to the loss of the glucose moiety, and the loss of 114 Da (fragment ion at *m/z* 221.0456 [M − H − 162 − 114] ^–^) was due to the loss of two aldehyde groups and cleavage of the substituent ring (28 Da), which was based on the hemiacetal group being easily converted into an epimeric isomer. Therefore, the hemiacetal group isomerised into two aldehyde groups. For these reasons, compound **1** was identified as verproside. Compounds **3 **−** 5** and **7 **−** 9** exhibited [M − H]^−^ ions at *m/z* 481.1327 (**3**; calcd for C_22_H_26_O_12_, 481.1346, *t_R_* = 4.94 min), 523.1436 (**4**; calcd for C_24_H_28_O_13_, 523.1452, *t_R_* = 5.99 min), 511.1451 (**5**; calcd for C_23_H_28_O_13_, 511.1452, *t_R_* = 6.12 min), 511.1447 (**7**; calcd for C_23_H_28_O_13_, 511.1452, *t_R_* = 6.52 min), 537.1647 (**8**; calcd for C_25_H_30_O_13_, 537.1608, *t_R_* = 8.69 min), and 623.1953 (**9**; calcd for C_24_H_30_O_13_, 623.1976, *t_R_* = 9.28 min). These compounds also showed fragment ion losses similar to those of verproside (**1**): glucose (−162 Da) and two aldehyde groups and cleavage of the substituent ring (−114 Da). Therefore, these compounds were identified as catalposide (**3**), verminoside (**4**), picroside II (**5**), isovanillyl catapol (**7**), minecoside (**8**), and 6-*O*-veratroyl catalpol (**9**). Compounds **2** (*m/z* 533.1028, calcd for C_22_H_27_ClO_13_, 533.1062, *t_R_* = 4.94 min) and **6 (***m/z* 533.1064, calcd for C_22_H_27_ClO_13_, 533.1062, *t_R_* = 6.23 min) were deduced from the quasimolecular 3:1 ion cluster, assuming that these compounds contained Cl, and were identified as longifolioside A (**2**) and piscroside C (**6**) (Table 1).

### Isolation and identification of the iridoids

**Figure 2.**
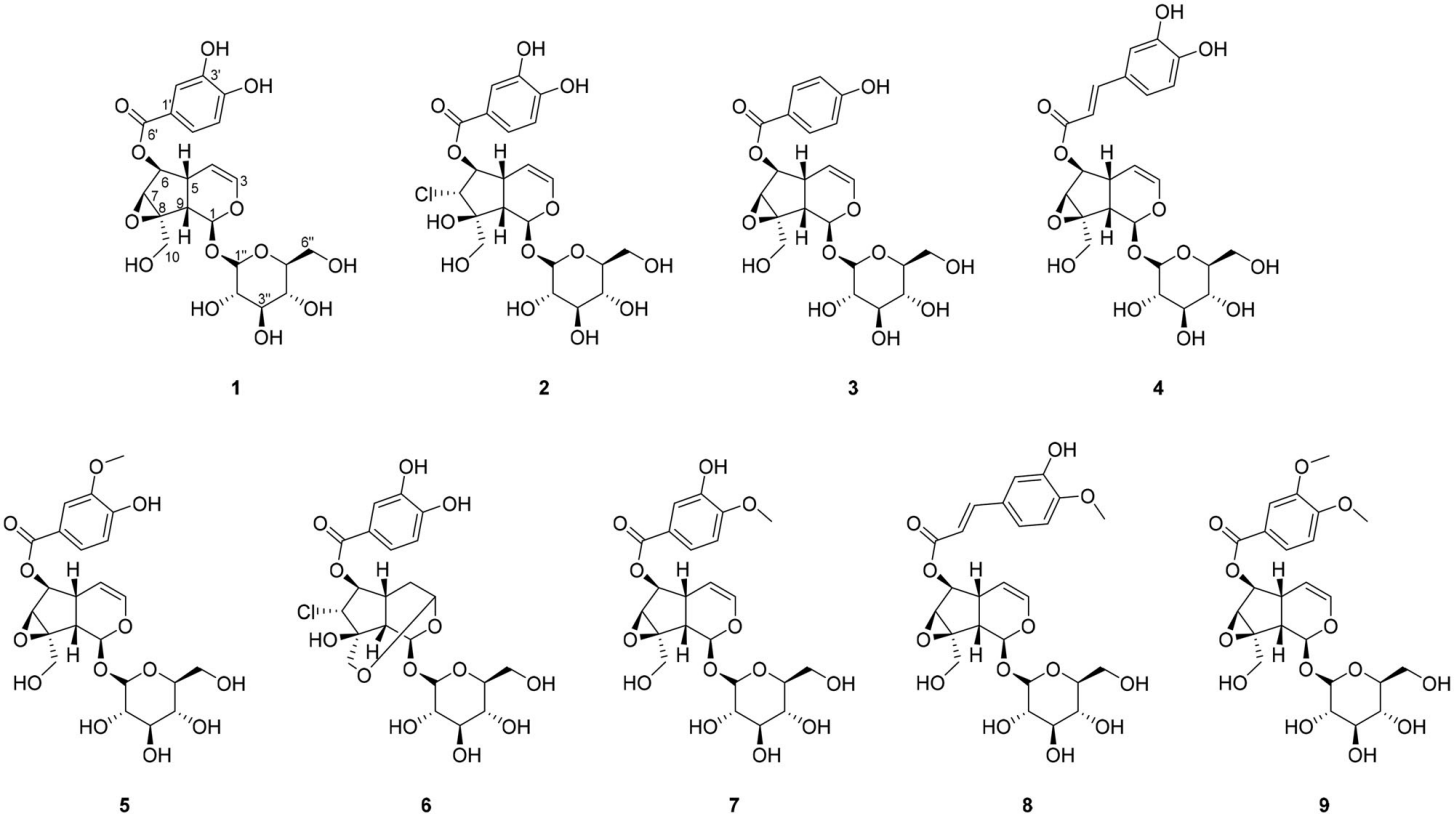
Chemical structures of the major compounds isolated from the extract of the whole plant of *Pseudolysimachion rotundum* var. subintegrum.

Nine compounds (**1 **−** 9**) were isolated from the whole plant of *P. rotundum* var. *subintegrum* and their identities were elucidated based on 1D and 2D NMR data. Most of the isolated compounds, except for **2** and **6**, from *P. rotundum* var. *subintegrum* were iridoid glycosides with an aglycone oxygen bridge between C-7 and C-8 with different benzene substituent groups at C-6 and a glucose linked to the *O*-aglycone at the C-1 position. The discussion of structural identification will focus on compound **1**, which emerged as the most potent tyrosinase inhibitor. Compound **1** was deduced to be a glucoside according to the general appearance of its ^1^H and ^13^C NMR spectra (Figures S1 and S2). The ^13^C NMR data showed 22 carbons, including five quaternary carbons, two methylene carbons and fifteen methine carbons. The ^1^H and ^13^C spectra suggested an iridoid-type skeleton with one glucoside unit [^13^C NMR: *δ*_C_ 98.3 (C-Glc-1″), 73.9 (C-Glc-2″), 76.9 (C-Glc-3″), 70.7 (C-Glc-4″), 77.9 (C-Glc-5″), 61.8 (C-Glc-6″); ^1^H NMR: *δ*_H_ 4.61 (d, *J* = 7.8 Hz, H-Glc-1″), 3.06 (d, *J* = 7.8 Hz, H-Glc-2″), 3.17 (m, H-Glc-3″), 3.02 (d, *J* = 8.8 Hz, H-Glc-4″), 3.14 (m, H-Glc-5″), 3.47 (dd, *J* = 6.8, 11.6 Hz, H-Glc-6″a), 3.72, (m, H-Glc-6″b)]. The ^1^H NMR signals from the aglycone moiety showed five aromatic/olefinic methines [*δ*_H_ 7.40 (d, *J* = 2.0 Hz, H-2′), 7.36 (dd, *J* = 2.0, 8.4 Hz, H-6′), 6.83 (d, *J* = 8.4 Hz, H-5′), 6.42 (dd, *J* = 1.6, 5.6 Hz, H-3), 4.94 (dd, *J* = 4.4, 5.6 Hz, H-4)], three oxygenated methines [*δ*_H_ 5.10 (d, *J* = 9.2 Hz, H-1), 5.04 (dd, *J* = 0.8, 8.0 Hz, H-6), 3.69 (d, *J* = 8.0 Hz, H-7)], an oxymethylene [*δ*_H_ 3.92 (d, *J* = 13.6 Hz, H-10a), 3.72 (d, *J* = 13.6 Hz, H-10b)], and two methines [*δ*_H_ 2.50 (m, H-5), 2.47 (d, *J* = 8.0 Hz, H-9)]. The ^13^C NMR revealed 16 carbon resonances, including a carbonyl carbon [*δ*_c_ 166.1 (C-7′)], three oxygenated aromatic/olefinic quaternary carbons [*δ*_C_ 151.3 (C-4′), 145.6 (C-3′), 120.4 (C-1′)], an oxygenated quaternary carbon [*δ*_C_ 66.2 (C-8)], five aromatic/olefinic methanes [*δ*_C_ 141.6 (C-3), 122.6 (C-5′), 116.8 (C-2′), 115.8 (C-6′), 102.2 (C-4)], three oxygenated methines [*δ*_C_ 93.4 (C-1), 79.9 (C-6), 58.9 (C-7)], an oxymethylene [*δ*_C_ 58.7 (C-10)], and two methines [*δ*_C_ 42.3 (C-9), 35.7 (C-5)]. The HMBC correlations from H-2′ to C-4′/C-6′/C-7′, H-5′ to C-1′/C-3′, and H-6′ to C-2′/C-4′/C-7′ revealed the presence of a dihydroxybenzoyl group, and the HMBC correlation from H-6 to C-7′ suggested that the dihydroxybenzoyl group was attached to C-6 in the structure of **1**. The glucose moiety was attached at C-1 based on the HMBC correlation from H-1 to C-Glc-1″. Therefore, the structure of compound **1** was deduced as shown in Figure 2 and identified as verproside. The NMR data of the other isolated compounds (**2 **−** 9**) were quite similar to those of verproside (**1**), except for the oxygen bridge between C-7 and C-8 and the substituent groups at C-6. The significant difference between **2** and verproside (**1**) was the presence of Cl at C-7 in **2** instead of an oxygen bridge between C-7 and C-8 based on the downfield shifts of C-7 (*δ*_C_ 69.1) and C-8 (*δ*_C_ 79.0). Compounds **3 **−** 5** and **7 **−** 9** differed by only their replacement of the 3,4-dihydroxybenzoyl group at C-6 with 4-hydroxybenzoyl, 3,4-dihydroxyphenyl, 3-methoxy-4-hydroxybenzoyl, 3-hydroxy-4-methoxybenzoyl, 3-methoxy-4-hydroxyphenyl, and 3,4-dimethoxybenzoyl groups, respectively. Compound **6** was most similar to **2**, containing an acetyl linkage between C-3 and C-10, as suggested from the HMBC correlation between H_2_-10 and C-3. Thus, compounds **2 **−** 9** were identified as longifolioside A (**2**), catalposide (**3**), verminoside (**4**), picroside II (**5**), piscroside C (**6**), isovanillyl catapol (**7**), minecoside (**8**), and 6-*O*-veratroyl catalpol (**9**), and the structures of these compounds are shown in Figure 2.

### Effects of iridoids on the activity of tyrosinase

**Figure 3.**
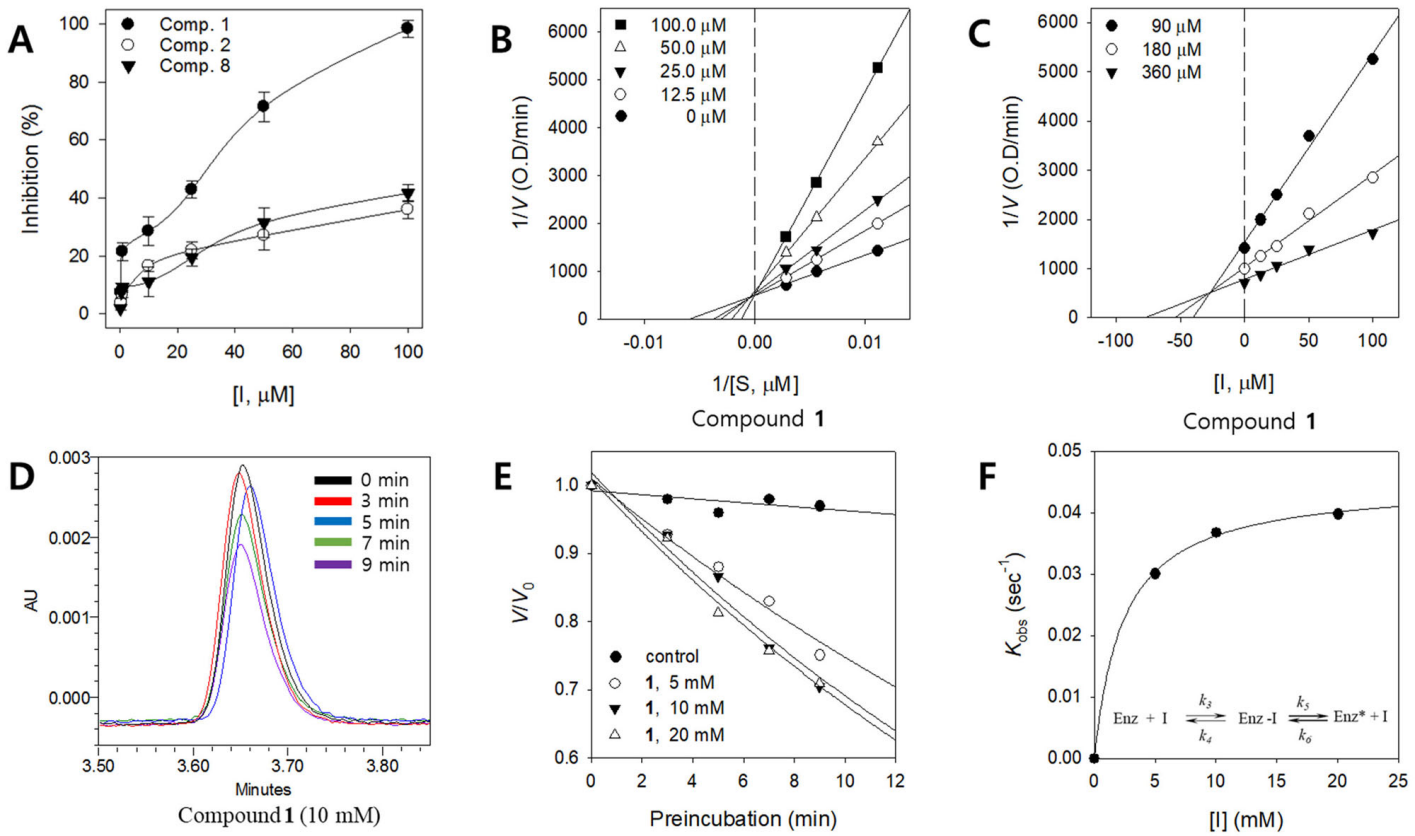
(A) Effects of compounds **1**, **2**, and **8** on the activity of tyrosinase to catalyse L-tyrosine. (B) Lineweaver-Burk plots to investigate the ability of compound **1** to inhibit the monophenolase activity of tyrosinase. (C) Dixon plots to investigate the ability of compound **1** to inhibit the monophenolase activity of tyrosinase. (D) Time-dependent chromatograms of tyrosinase in the presence of compound **1** using UPLC-PDA. (E) Inhibition as a function of preincubation time for the most active compound **1** (5, 10, and 20 mM). (F) Plot of *k*_obs_ as a function of the concentration of the slow-binding inhibitor **1** fitted by Equation (4).

In the preliminary screening, we observed that iridoids from the whole plant of *P. rotundum* var. *subintegrum* showed significant inhibition of L-tyrosinase oxidation. Therefore, more detailed bioassays of the isolated compounds were subsequently conducted. The isolated compounds showed a dose-dependent inhibitory effect on monophenolase activity (mTyr). As the concentrations of the inhibitors increased, the residual enzyme activity was drastically diminished (Figure 3A). As shown in Table 2, all the iridoids investigated, especially compounds **1**, **2**, and **8**, exhibited significant dose-dependent inhibition of monophenolase (which uses L-tyrosine as a substrate) (IC_50_ values of 31.2, 192.9 and 131.8 μM, respectively). The potency of verproside (**1**, IC_50_ = 31.2 μM) can be favourably compared with commercially available inhibitors currently used as cosmetics, such as kojic acid (IC_50_ = 14.8 μM). Based on the above results, the simplest and most effective strategy is determine tyrosinase inhibitory effect agents that can reduce melanin production. However, most of the inhibitors mentioned in the literature have been tested and reported by mTyr^2^^,^^3^^,^^5^^,^^7^^,^^9^^,^^15^^,^^23^^,^^24^. Therefore, tyrosinase inhibitory activity has been reported in relation to the structural characteristics of natural plant inhibitors with a hydroxyl groups and synthetic compounds based on natural products^36^^,^^37^. Specifically, Ahmed’s group investigated the effects of natural products and their derivatives on the activity of mushroom tyrosinase, such as dihydro-1,5-benzothiazepines (IC_50_ = 1.21–70.65 µM)^38^, flavone-based hydrazones (IC_50_ = 0.95–4.12 µM)^39^, thioflavones, and thioflavonols (IC_50_ = 1.12–5.68 µM)^40^. Another study examined the effects of aurone derivatives (IC_50_ = 7.12–66.82 µM)^41^ to discover potent tyrosinase inhibitors. Therefore, we are confident that the previously studied compounds, including iridoid glycosides, are leading structures for the design and development of new tyrosinase inhibitors. Most of the inhibitors mentioned in the literature have been tested and reported by mTyr and inevitably lack clinical efficacy when incorporated into products. Thus, by retesting in hTyr, a strategy was established to discover inhibitors that are effective in mTyr and are clinically accessible. Next, the most potent compound, verproside (**1**), was analysed for its ability to inhibit hTyr, and the results were compared using kojic acid as the positive control group or the inhibitory effects value of the previously literature. Verproside (**1**) also showed a dose-dependent inhibitory effect on hTyr, with an IC_50_ value of 197.3 μM, which was 5.6 times lower than that of the positive control kojic acid. Furthermore, by comparing previously literature for hTyr, it was established that kojic acid produced a more effective inhibitor (**1**, IC_50_ = 197.3 μM, kojic acid IC_50_ = 1109.3 μM vs IC_50_ = 117.0 μM)^42^. In addition, verproside did not significantly affect cell viability in B16F10 and Malan-A cells (Figure S4).

**Table 2.** Inhibitory effects of isolated iridoids **1 **−** 9** on tyrosinase activity.

Compounds	Mushroom Tyrosinase L-Tyrosine			Mushroom Tyrosinase L-DOPA	Human Tyrosinase L-Tyrosine	Human Tyrosinase L-DOPA
	IC_50_ (µM)^a^	100 µM inhibition (%)	Type of inhibition (*K*_i_, µM)^b^	IC_50_ (µM)^a^	IC_50_ (µM)^a^	IC_50_ (µM)^a^
Verproside (**1**)	31.2 ± 2.1	98.4	Competitive (5.1 ± 0.7)	177.2 ± 33.1	197.3	842.7
Longifolioside A (**2**)	192.9 ± 10.5	36.0	NT	> 200	NT	NT
Catalposide (**3**)	>200	9.9	NT	> 200	NT	NT
Verminoside (**4**)	>200	8.9	NT	> 200	NT	NT
Picroside II (**5**)	>200	5.3	NT	> 200	NT	NT
Piscroside C (**6**)	>200	6.7	NT	> 200	NT	NT
Isovanily catapol (**7**)	>200	10.1	NT	> 200	NT	NT
Minecoside (**8**)	131.8 ± 8.3	41.7	NT	> 200	NT	NT
6-*O*-Veratroyl catalpol (**9**)	>200	6.1	NT	> 200	NT	NT
Kojic acid^d^	14.8 ± 0.6	NT	NT	37.1 ± 1.3	1109.3	1819.7
					(117.0)^e^	(185.0)^e^

All compounds were examined in a set of experiments repeated three times

^a^IC_50_ values of compounds represent the concentration that caused 50% enzyme activity loss

^b^Values of inhibition constant.

^c^NT is not test.

^d^Positive control.

^e^Monophenolase activity 117 µM and diphenol oxidase activity 185 µM IC_50_ value calculated from Dolinska et al., 2014.^36^

Subsequently, full characterisation of the kinetic behaviour of the oxidation of L-tyrosine catalysed by tyrosinase in the presence of verproside (**1**) was investigated. In this experiment, the initial velocity of the enzyme was monitored by observing dopachrome formation at 475 nm. Verproside (**1**) displayed competitive inhibition against both monophenolase (*K*_i_ = 5.1 μM) and diphenolase activity (*K*_i_ = ND), as shown in the Dixon and Lineweaver-Burk plots, respectively (Figure 3(B–C)). Most competitive inhibitors of tyrosinase bind to the active site, mimicking the enzyme/substrate interaction. The B-ring of our isolated compounds is very similar to the tyrosinase substrate tyrosine). This leads to the competitive displacement of the substrate from the active site with the cofactor via the lock-and-key model. Studies have found that some compounds can also chelate copper, which has been suggested as a mode of inhibition.

Most competitive inhibitors act with slow binding to the enzyme active site. To determine the mode of inhibition of a time-dependent inhibitor, it is convenient to analyse the effect of various substrate concentrations on *k*_obs_ at a fixed inhibitor concentration. A competitive inhibitor will display a decrease in *k*_obs_ with increasing substrate concentration. Mushroom tyrosinase showed time-dependent inhibition in the presence of verproside (**1**). Increasing verproside (**1**) concentration led to a decrease in both the initial velocity (*v*_i_) and the steady-state rate (*v*_s_) in the progress curve. The progress curves obtained using various concentrations of the inhibitors were fitted to Equation (2) to determine *v*_i_, *v*_s_, and *k*_obs_. The binding event was assessed using an experimental setup involving an initial incubation step [5 mM verproside (**1**) with 144 U of enzyme], followed by centrifugation and finally analysis of the flow-through (i.e. the fraction not bound by the enzyme) using UPLC-PDA rather than a UV–vis spectrophotometer (Figure 3D). Therefore, Equation (2) could not be used, but Equations 3 and 4 could be used with the following step plots. The enzyme was preincubated with inhibitor for a duration of between 0 and 9 min, and the velocity of the reaction was measured as a function of incubation time and analysed according to Equation (3) (Figure 3E). Such behaviour is typical of a slow-binding inhibitor. In Figure 3(F), the data were fitted to Equation (4) and showed a hyperbolic dependence (*R*^2^ = 0.9994) on the concentration of inhibitor **1**, so inhibition by **1** is believed to follow the mechanism of the enzyme isomerisation model. The *y* intercept of the curve provides an estimate of the rate constant *k*_6_, while the maximum value of *k*_obs_ is expected at infinite inhibitor concentrations. Equation (4) also allowed calculation of the kinetic parameters *k*_5_, *k*_6_ and *K*^app^_i_. Thus, analysis of the data according to Equation (4) yielded the following values: *k_5_* = 0.04496 s^−^^1^, *k*_6_ = 0.00001103 s^−^^1^, and *K*_i_^app^ = 2.402 mM. These results indicated that verproside (**1**) inhibits tyrosinase by rapidly forming an enzyme–substrate complex (Enz-I), which slowly isomerises to form a modified enzyme complex (Enz*-I).

### Computational docking simulations to determine the tyrosinase ligand candidates

Docking simulations were performed with the nine compounds in all existing tyrosinase pockets (details are included in the Methods). As shown in Table 3, the docking results of the nine compounds are tabulated by the lowest energy, the number of conformations in a group, the average energy in a group, and the binding location. The experimental results show that verproside (**1**) has meaningful IC_50_ values (Table 2) via competitive inhibition; i.e. it directly inhibits the active site of tyrosinase. Verproside (**1**) was docked into the active site of tyrosinase with the lowest energy of −6.9 kcal/mol and the highest number of conformations at 43. Longifolioside A (**2**) and minecoside (**8**) had the lowest binding energies of −6.6 and −6.2 kcal/mol with numbers of 30 and 31, respectively. These numbers are larger than those of verminoside (**4**), picroside II (**5**), piscroside C (**6**), and 6-*O*-veratroyl catalpol (**7**). The magnitude of the number indicates how frequently the compound bound to the active site irrespective of the energy. The binding poses of the compounds in the active site, including the two abovementioned compounds, are shown in Supplementary material Figure S5.

**Table 3.** Docking simulation results of isolated iridoids **1 **−** 9** on tyrosinase activity.

Compounds	Lowest energy (kcal/mol)	Number in group^a^	Average energy in group (kcal/mol)	Binding location^b^
Verproside (**1**)	−6.9	43	−3.72	Active
Longifolioside A (**2**)	−6.6	30	−5.04	Other
Catalposide (**3**)	−7.2	44	11.36	Active
Verminoside (**4**)	−6.6	24	−5.07	Other
Picroside II (**5**)	−6.7	29	−1.91	Other
Piscroside C (**6**)	−7.2	29	−4.64	Active
Isovanillyl catapol (**7**)	−6.3	48	4.82	Other
Minecoside (**8**)	−6.2	31	6.63	Other
6-*O*-Veratroyl catalpol (**9**)	−6.2	24	−4.60	Other

^a^The number of compounds existing in the same pocket.

^b^Active: a compound docks on the tyrosinase active site.

Other: a compound docks on the other side which is not related on the enzyme reaction.

**Figure 4.**
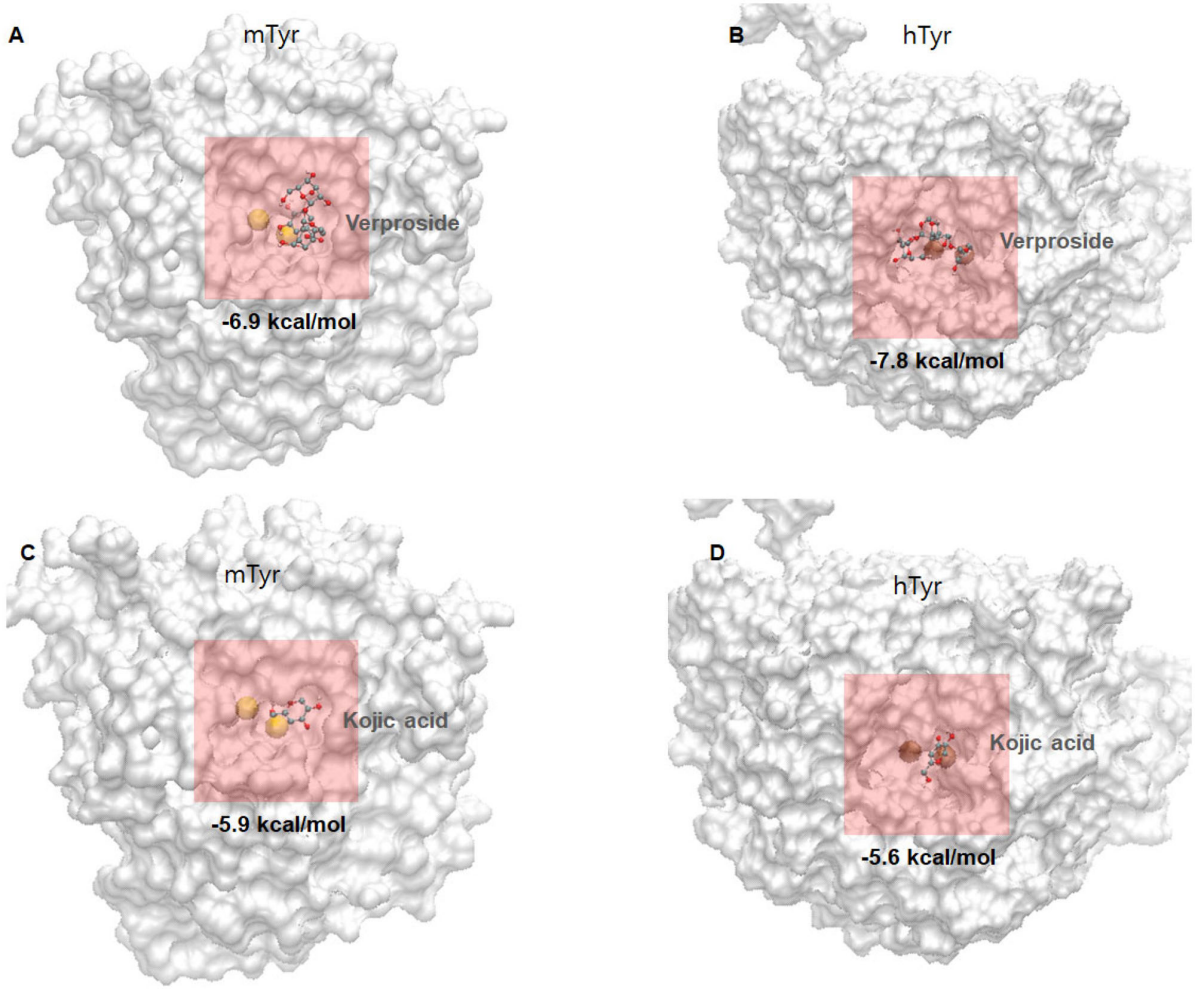
Molecular docking views of two compounds, verproside and kojic acid, on two tyrosinases: (A) verproside-mTyr, (B) verproside-hTyr, (C) kojic acid-mTyr, and (D) kojic acid-hTyr. The tyrosinases are represented by a white surface protein model, and the two Copper ions present in the tyrosinases are represented by a yellow space-filling model. The active site is highlighted by a red transparent box. The compounds are represented by ball and stick models with atom colours: red for Oxygen, cyan for Carbon, and white for Hydrogen.

**Figure 5.**
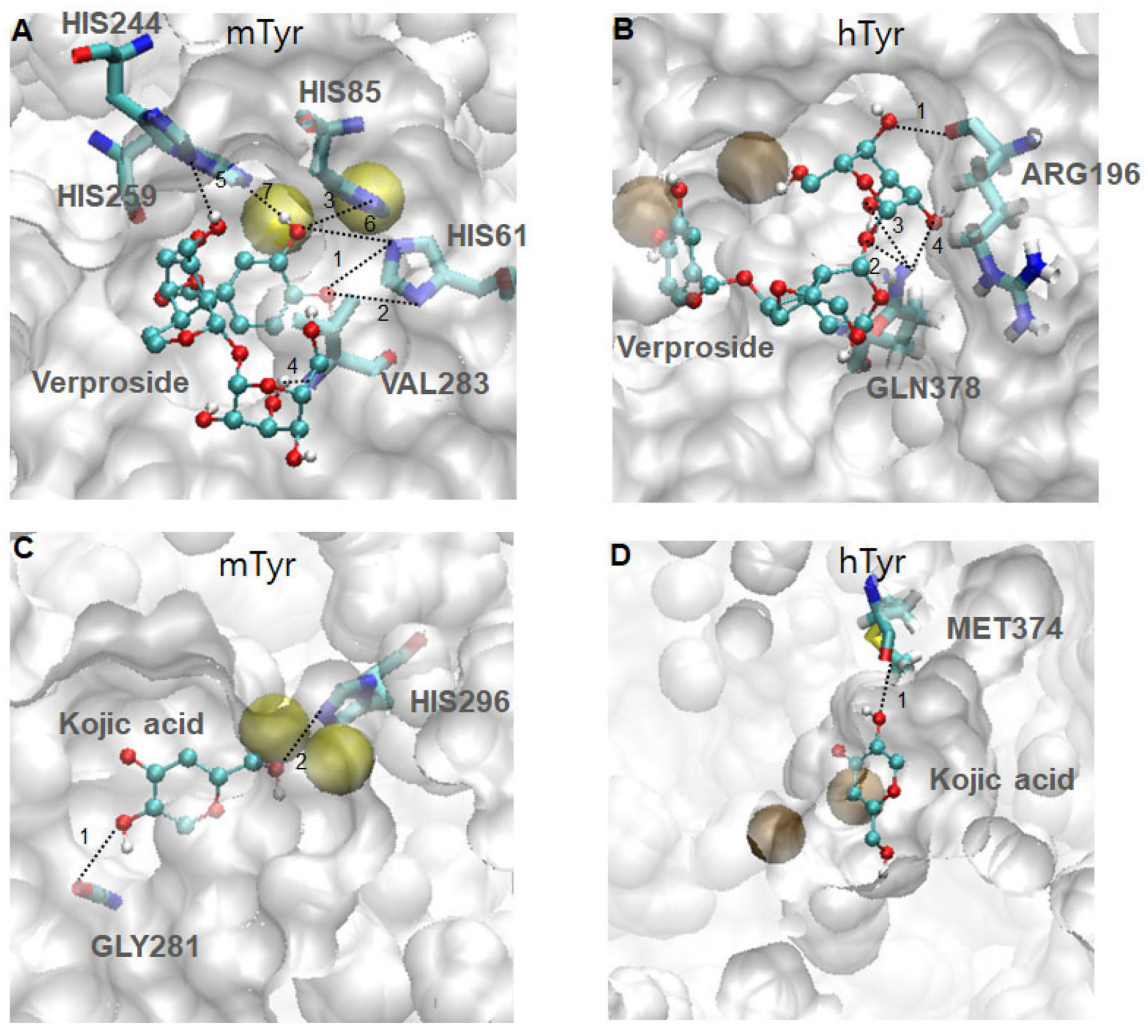
Hydrogen bonding patterns of two compounds, verproside and kojic acid, on two tyrosinases, mTyr and hTyr: (A) verproside-mTyr, (B) verproside-hTyr, (C) kojic acid-mTyr, and (D) kojic acid-hTyr. The active site, highlighted by the red transparent box in Figure 4, is magnified to show the detailed hydrogen bonding pattern. The compounds are drawn using the ball and stick model and coloured by atom: red for Oxygen, cyan for Carbon, and white for Hydrogen. The hydrogen bonding interactions are indicated by dotted lines with the interaction numbers. The detailed hydrogen bonding patterns are provided in Supplementary material Table S2.

The docking results were consistent with the experimental results for verproside (**1**). Therefore, further analysis of verproside (**1**) was carried out. Figure 4(A) shows the verproside (**1**) docking pose with tyrosinase. Verproside (**1**) was well docked into the active site with a docking energy of −6.9 kcal/mol. The dihydroxy group in the phenyl ring (the upper ring in Figure 2) penetrates the active site pocket, interacts with the two Copper ions and forms hydrogen bond with the acceptor atoms of two amino acids, HIS61 and HIS85. As shown in Figure 5(A), seven hydrogen bonds were identified. Detailed information on these hydrogen bonds, such as the donors, acceptors, and distances, is tabulated on Supplementary material Table S2.

The computational results in Table 3 show that catalposide (**3**) and piscroside C (**6**) also bind to the active site of tyrosinase. However, compared with verproside (**1**), these two compounds showed different results. Although both have lower binding energies (i.e. they bind more tightly) than verproside (**1**), catalposide (**3**) displayed frequent binding with a score of 44, but had a large average energy in the group of 11.36 kcal/mol, whereas piscroside C (**6**) had a small occupancy number (29) and an average energy of −4.64 kcal/mol. First, we investigated the results of catalposide (**3**). Although catalposide (**3**) has the lowest energy in the active site at −7.2 kcal/mol, and a large occupancy number in the group at 44, the average energy in the group had the positive value of 11.36 kcal/mol. In the structural view in Figure 2, catalposide (**3**) has one hydroxyl group on its phenyl ring. Based on the observations with verproside (**1**), this hydroxyl group makes important hydrogen bonding interactions with tyrosinase. The fewer hydroxyl functional groups in catalposide (**3**) than in verproside (**1**) (with two hydroxyl groups) results in a weak interaction and a larger average energy than verproside (**1**). Second, we examined piscroside C (**6**). Piscroside C (**6**) was inferred to have a smaller number than verproside (**1**) because of steric hindrance. As shown in Figure 2, piscroside C (**6**) has an epoxy linker (-*O*-) in the centre ring, and this linker could interrupt binding to the active site; thus, some poses can bind to tyrosinase while the others fail to bind. Additionally, piscroside C (**6**) has a chlorine in the centre ring, which is a strong electron withdrawing group. Because of the steric hindrance from the linker and electron withdrawing group, piscroside C (**6**) has a smaller occupancy number in the active site than verproside (**1**). Although piscroside C (**6**) gave lower energy than verproside (**1**), the smaller number in the group makes piscroside C (**6**) binding unfavourable. In summary, among the three compounds, verproside (**1**), catalposide (**3**), and piscroside C (**6**), with the lowest energy poses in the active site, verproside (**1**) is the most potent inhibitor when energetic and structural factors are considered.

From a structural perspective, verproside (**1**) is a potent inhibitor of tyrosinase, whereas the other investigated compounds contain weak points in their structures. Longifolioside A (**2**) and piscroside C (**6**) have chlorine atoms in the centre ring, which is a strong electron withdrawing group (Figure 2). Catalposide (**3**), picroside II (**5**), isovanillyl catapol (**7**), minecoside (**8**), and 6-*O*-veratroyl catalpol (**9**) have one hydroxyl group on the upper phenyl ring, enabling fewer hydrogen bonds in the active site than verproside (**1**), which contains two hydroxyl groups. Verminoside (**4**) and minecoside (**8**) possess an ethylene double bond between the upper phenyl ring and the centre ring. Because this double bond is in the *trans*-conformation, the dihydroxy phenyl ring pointed towards the active site makes penetration difficult. Piscroside C (**6**) has an epoxy linker, giving the structure another ring in the centre ring. This produces large steric effects, and as a result, the active site is difficult to penetrate, leading to fewer interactions with the active site.

To clearly differentiate tyrosinases in mushrooms and humans, we will refer to them as mTyr and hTyr, respectively. As described in the Methods section, we obtained the structure of hTyr from the alpha fold database since its experimental structure has not been revealed yet. Among several compounds tested, verproside showed a high affinity for mTyr and was selected for further simulations. Kojic acid (Pubchem CID: 3840) is a well-known reference inhibitor of tyrosinases. We compared the docking results of verproside and kojic acid on both tyrosinases (mTyr and hTyr). Before comparing the docking results, we compared the structures of the two tyrosinases. As illustrated in Supplementary material Figure S6, we superimposed the two structures, and a backbone similarity of 4.2 Å was revealed. Although the two structures are quite similar, hTyr has a larger active pocket located near two Copper ions and more coiled structures.

In Figure 4, we present the four docking structures of both compounds on both tyrosinases, and the docking results are summarised in Table 4. All four simulations were well-docked near the active site, which is located near two Copper ions. The lowest docking score of the four simulations was −7.8 kcal/mol for verproside on hTyr, followed by verproside on mTyr with an energy of −6.9 kcal/mol. These results indicate that compared to kojic acid, verproside binds more tightly and is a competent inhibitor. Although verproside is more tightly bound to hTyr than mTyr in terms of energy values, its bound form on mTyr ranked first in the conformation group, and it exhibited larger hydrogen bonds than those on hTyr. The detailed hydrogen bond patterns of the four docking simulations are shown in Figure 5. Our results demonstrate that verproside is more tightly bound to mTyr than hTyr, which agrees with the experimental IC_50_ values (31.2 and 197.3 μM). Kojic acid has less energy than verproside because it is smaller and has fewer interactions, particularly hydrogen bonding interactions. Kojic acid on hTyr has the highest energy (-5.6 kcal/mol) of the four simulations due to its low hydrogen bonding (only one hydrogen bond) and energy rank, which is third in the conformation group. This is consistent with the experimental results, which showed an IC_50_ of 1109.3 μM.

**Table 4.** Docking simulation comparisons of verproside and kojic acid on mushroom (mTyr) and human tyrosinase (hTyr).

	IC_50_ (μM)		Docking energy^a^ (rank in group)		Number of Hydrogen bond^b^	
	mTyr	hTyr	mTyr	hTyr	mTyr	hTyr
Verproside	31.2	197.3	−6.9 (1)	−7.8 (2)	7	4
Kojic acid	14.8	1109.3	−5.9 (1)	−5.6 (3)	2	1

^a^Energy unit: kcal/mol

^b^The detail hydrogen bond patterns are shown on Table S2.

**Figure 6.**
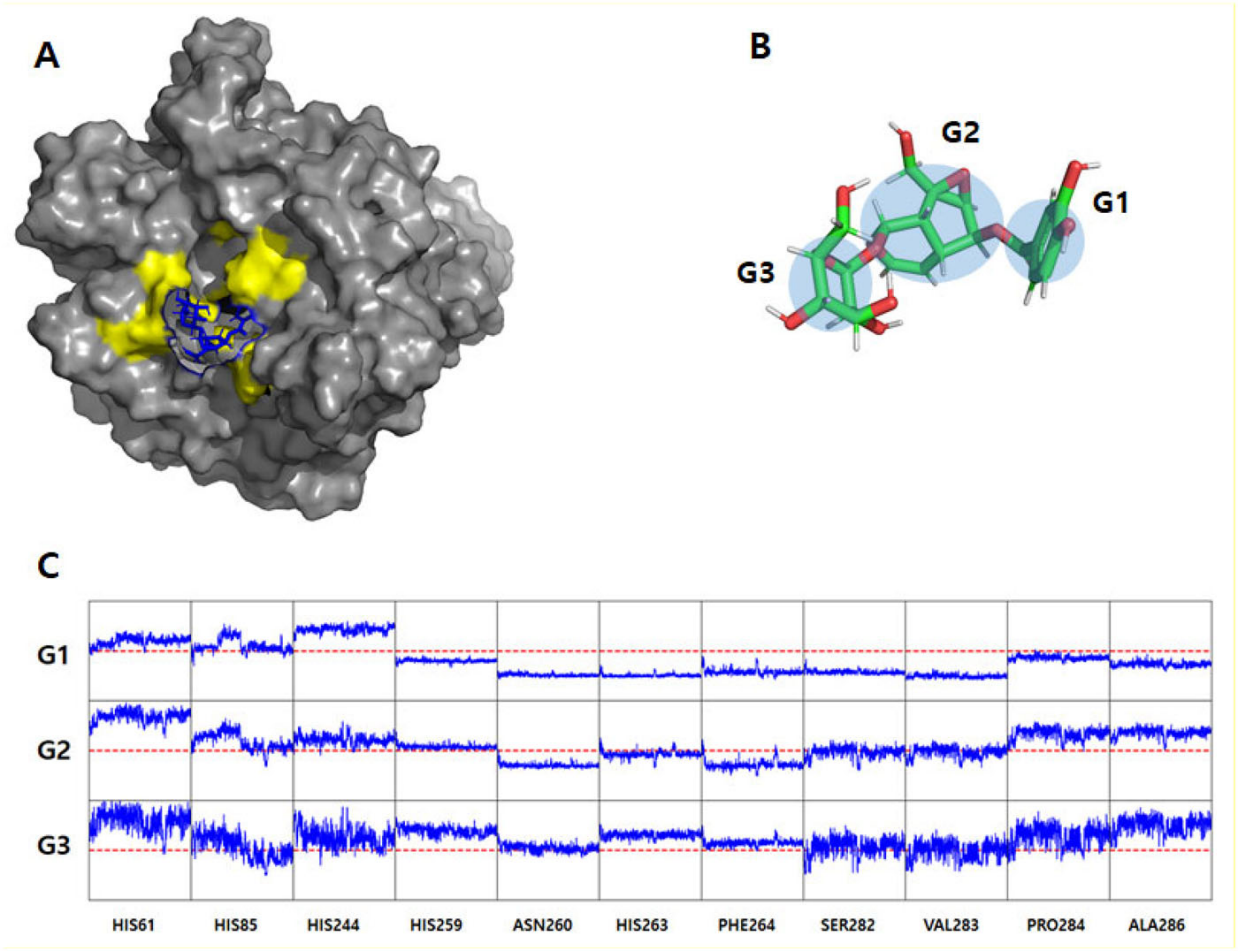
Molecular dynamics simulation results. (A) The final structures of mTyr-verproside complex. The tyrosinases are depicted using a grey surface model, while the active site revealed by docking simulation is coloured yellow. The verproside is represented by a blue stick model. For clarity, the explicit water models and counter ions are not included in the figure. (B) Verproside grouping. The centre of geometry (COG) of the atoms comprising the rings was utilised to measure the interacting distance. The measurement was conducted between the COG and the alpha Carbon atom of the interacting residues. (C) The interaction distance between verproside and mTyr. It consists of 33 sub-panels, each displaying the distance-time profile (X-axis: time from 0 to 100 ns; Y-axis: distance from 0 to 20 Å). The Y panel is divided into three groups, namely G1, G2, and G3, as mentioned in Figure 6B. The red dotted line represents the distance of 10 Å, which serves as the lower limit distance.

We performed MD simulations for two potent derivatives of verproside on mTyr and hTyr proteins. Figure 6 depicts the final structures obtained from 100-nanosecond (ns) explicit water molecular dynamics simulations. As shown in Figure 6(A), the verproside on mTyr remains bound to the active site, consistent with the docking simulation results. However, the verproside on hTyr dissociates from the active site and moves independently in proximity to the protein (Supplementary material Figure S7). To perform a detailed analysis, we measured the interaction distance between verproside and the key residues suggested by the docking simulation during the MD simulation. As the verproside consists of three rings, we divided it into three groups, as shown in Figure 6(B), to observe which parts remain fixed in the active site and which parts began to enter the pocket. Figure 6(C) illustrates that Group 1 (G1) serves as the anchor region of the verproside. It tightly interacts with HIS259, ASN260, HIS263, PHE264, SER282, VAL283, PRO284, and ALA286 within the pocket. These distances remain stable with minimal fluctuations, indicating that the binding is well-organised. G2 also maintains a well-packed position within the pocket. However, G3 shows weaker binding to some residues, and the distances are significantly greater (over 10 Å) from the pocket. In summary, the interaction between verproside and the protein’s residues corresponds well with the results obtained from the docking simulation. The distances are well-maintained, ensuring tight binding to the active site. Notably, G1 serves as the anchor region for inhibiting mTyr, and the key residues show a strong match on the docking results. However, the opposite trend was observed for the results obtained with verproside and hTyr (Supplementary material Figure S7). Experimental data reveals that hTyr-verproside exhibits higher IC_50_ values, indicating weaker binding affinity. Even though the docking simulation suggested that verproside on hTyr is a potent inhibitor of hTyr, the subsequent MD simulation revealed that verproside exhibits weaker binding to hTyr, which aligns well with the experimental findings. As part of the MD simulation validations, we assessed Root Mean Square Deviation (RMSD) and Radius of Gyration (Rgyr) over time, as well as Root Mean Square Fluctuation (RMSF) as a function of residue number. Supplementary material Figure S8 demonstrates that both tyrosinases maintained their structural integrity throughout the simulation.

In summary, the structure–activity studies revealed that iridoids from *P. rotundum* var. *subintegrum* are potent competitive inhibitors with a chemospecific inhibition mechanism. Verproside (**1**), which possesses two hydroxyl groups on the upper phenyl ring, worked through the enzyme isomerisation model with slow binding.

## Conclusion

The current experiments evaluating enzyme binding affinity were used to tentatively identify inhibitors of a particular enzyme because an enzyme–inhibitor complex must be formed for inhibition to occur. Thus, these iridoids inhibit tyrosinase activity through multiple mechanisms, which are mainly dependent on the hydroxyl group(s) on the upper phenyl ring and the C-7 substituent. Among these, dramatic differences in the binding affinity, docking simulations and IC_50_ values were observed, with the most notable compound being **1** (active site = 43, IC_50_ = 31.5 µM). Verproside-mediated inhibition of tyrosinase is reversible, but this interaction also disrupts the active site competitively, and verproside also interacts with the hydrophilic portion of the enzyme surrounding the active site. The inhibition kinetics analysed by Lineweaver-Burk plots indicated that verproside is a competitive, slow-binding inhibitor, and the *K*_i_ was determined to be 5.1 μM. Thus, verproside (**1**) is a competitive inhibitor with a chemo-specific inhibition mechanism that operates via the enzyme isomerisation model. Additional well-known tyrosinase hyperactivity inhibitors include compounds such as hydroquinone (mutagenic, dermatitis, and irritation), arbutin (unstable), L-ascorbic acid (degradation), kojic acid (carcinogenic), and ellagic acid (solubility). However, these inhibitors exhibit many side effects. Therefore, the plant-derived tyrosine inhibitors, verproside, studied herein displayed the most potent inhibition profiles, suggesting that these inhibitors are excellent therapeutic candidates to prevent excessive melanin production and accumulation for the treatment of melasma, freckles, ephelides, and solar lentigines.

## Supplementary Material

Supplemental Material
