# Supplementary material for: Potent inhibition of human tyrosinase inhibitor by verproside from the whole plant of Pseudolysimachion rotundum var. subintegrum
Source: J Enzyme Inhib Med Chem. 2023 Aug 30;38(1):2252198. doi: 10.1080/14756366.2023.2252198 (PMC10472861; doi:10.1080/14756366.2023.2252198)
Supplement: Supplemental Material [file IENZ_A_2252198_SM3722.pdf]

Potent inhibition of human tyrosinase inhibitor by verproside from the whole plant of *Pseudolysimachion rotundum* var. *subintegrum*

Sunin Jung [a1], So-Yeun Woo [a,1], Mi Hyeon Park [a,1], Doo-Young Kim [a], Su Ui Lee [a], Sei-Ryang Oh [a], Mun-Ock Kim [a], Jinhyuk Lee [b,c *], Hyung Won Ryu [a,*]

[a] Natural Medicine Research Center, Korea Research Institute of Bioscience and Biotechnology, Cheong-ju si, Chungcheongbuk-do 28116, Republic of Korea

[b] Disease Target Structure Research Center, Korea Research Institute of Bioscience and Biotechnology (KRIBB), Gwahak-ro, Yuseong-gu, Daejeon 34141, Republic of Korea

[c] Department of Bioinformatics, KRIBB School of Bioscience, University of Science and Technology (UST), 217 Gajung-ro, Yuseong-gu, Daejeon 34113, Republic of Korea

Running title: Competitive binding inhibitors of tyrosinase from *P. rotundum*

* Corresponding authors. tel. +82-42-879-8530 (J.H. Lee), tel. +82-43-240-6117; fax +82-43-240-6119 (H.W. Ryu).

E-mail address: E-mail: jinhyuk@kribb.re.kr (J.H. Lee), ryuhw@kribb.re.kr (H.W. Ryu).

[1] These authors contributed equally to this work.

Table of Contents

Figure S1-1 Preparative HPLC fractionation of *P. rotundum* var. *subintegrum* extract 3

Figure S1-2. UPLC-PDA of fractions of *P. rotundum* var. *subintegrum* extract...4

Figure S2-1. ^{1}H and ^{13}C NMR spectrum of Verproside (**1**) 5

Figure S2-2. ^{1}H and ^{13}C NMR spectrum of Longifolioside A (**2**)...6

Figure S2-3. ^{1}H and ^{13}C NMR spectrum of Catalposide (**3**)..7

Figure S2-4. ^{1}H and ^{13}C NMR spectrum of Verminoside (**4**) 8

Figure S2-5. ^{1}H and ^{13}C NMR spectrum of Picroside II (**5**) 9

Figure S2-6. ^{1}H and ^{13}C NMR spectrum of Piscroside C (**6**) 10

Figure S2-7. ^{1}H and ^{13}C NMR spectrum of Isovanillyl catalpol (**7**) 11

Figure S2-8. ^{1}H and ^{13}C NMR spectrum of Minecoside (**8**)...12

Figure S2-9. ^{1}H and ^{13}C NMR spectrum of 6-*O*-Veratroyl catalpol (**9**)...13

Figure S3-1. UV, MS/MS, MS and HREIMS data of Verproside (**1**) 14

Figure S3-2. UV, MS/MS, MS and HREIMS data of Longifolioside A (**2**) 14

Figure S3-3. UV, MS/MS, MS and HREIMS data of Catalposide (**3**) 15

Figure S3-4. UV, MS/MS, MS and HREIMS data of Verminoside (**4**)...15

Figure S3-5. UV, MS/MS, MS and HREIMS data of Picroside II (**5**)...16

Figure S3-6. UV, MS/MS, MS and HREIMS data of Piscroside C (**6**) 16

Figure S3-7. UV, MS/MS, MS and HREIMS data of Isovanillyl catalpol (**7**)................................17

Figure S3-8. UV, MS/MS, MS and HREIMS data of Minecoside (**8**)...17

Figure S3-9. UV, MS/MS, MS and HREIMS data of 6-*O*-Veratroyl catalpol (**9**) 18

Figure S4. The cell viability by verproside in B16F10 and Melna-A cells.....................................19

Figure S5. Structural views of nine compounds on the active site of mushroom tyrosinase...........20

Figure S6. Structural comparisons on mushroom (mTyr) and human tyrosinases (hTyr). 21

Figure S7. Molecular dynamics simulation results. 22

Figure S8. The structure validation during MD simulation for mTyr (left) and hTyr (right) 23

Table S1. The method conditions of ultrafiltration samples...24

Table S2. Hydrogen bonding pattern of Verproside and Kojic acid on mushroom (mTyr) and human tyrosinases (hTyr)...25

Experimental Section

1D (^{1}H, and ^{13}C) NMR spectra were obtained on JEOL ECZ500R (^{1}H NMR at 500 MHz, ^{13}C NMR at 125 MHz, Tokyo, JP), Varian UNITY 400 NMR(^{1}H NMR at 400 MHz, ^{13}C NMR at 100 MHz, PaloAlto, USA) and Bruker AVANCE III HD 700 (^{1}H NMR at 700 MHz, ^{13}C NMR at 175 MHz) using acetnone-d_6 (Cambridge Isotope Laboratories, Andover, MA) as an NMR solvent and tetramethylsilane (TMS) as an internal standard. HRESIMS were measured on an ultraperformance liquid chromatography quadrupole time-of-flight mass spectrometer (UPLC-QTof-MS, Waters, Milford, MA, USA) in the negative-ion mode. Preparative HPLC was performed with a K-Prep LAB-300G instrument (YMC, Kyoto, Japan). Semiprepartive HPLC separation (Gilson, Middleton, WI, USA) is comprised of a standard binary pump (321 HPLC pump), UV/Vis detector (172 DAD), evaporative light-scattering detector (ELSD, Varian 380-LC), and injection modules (GX271 liquid handler).

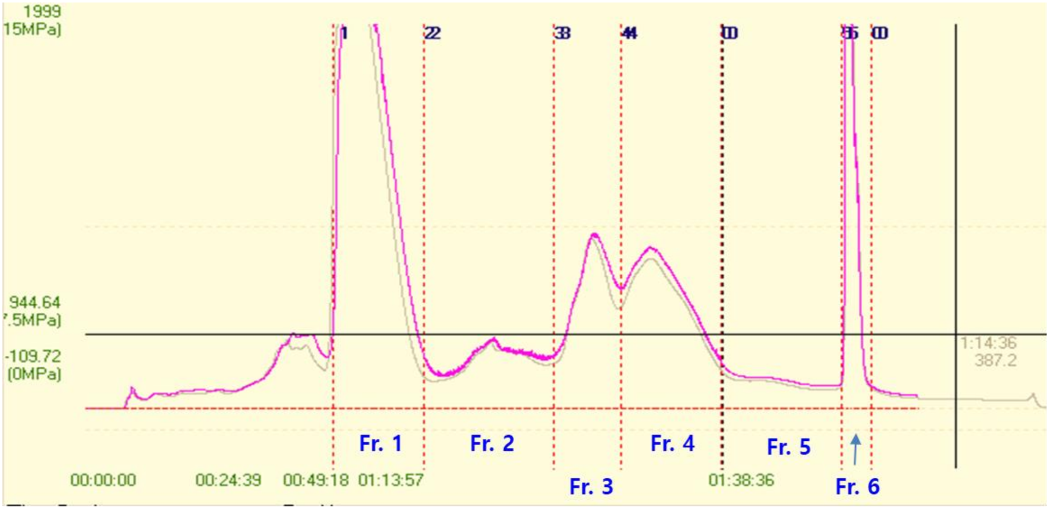

Condition

YPL_001	
Instrument	YMC_LAB
Guard Column	YMC-ODS-AQ-HG-10 um, 298 g<br>YMC-ODS-AQ-HG-10 um, 220 g
Solvents	MeOH/D.W
Flow Rate	150 ml/min
UV	254 nm
Loading sample	4 g/9 ml

Method - Gradient

Time(min)	%A	%B
(Initial)	73	27
10.00	73	27
60.00	65	35
61.00	0	100
76.00	0	100
77.00	73	27
97.00	73	27

Figure S1-1. Preparative HPLC fractionation of *P. rotundum* var. *subintegrum* extract.

UPLC method

① UPLC

Waters Acquity UPLC system

② Column

ACQUITY UPLC ® BEH C18 1.7㎛ 2.1x100mm

③ Absorbance

254nm

④ Concentration : 1mg/ml

⑤ Injection volume : 3 µl

⑥ Gradients

Time(min)	Flow(mL/min)	%A(0.1% Formic acid/DW)	%B(0.1% Formic acid/ACN)
0.00	0.4	90	10
1.00	0.4	90	10
10.50	0.4	77	23
12.00	0.4	2	98
13.30	0.4	2	98
13.40	0.4	90	10
15.00	0.4	90	10

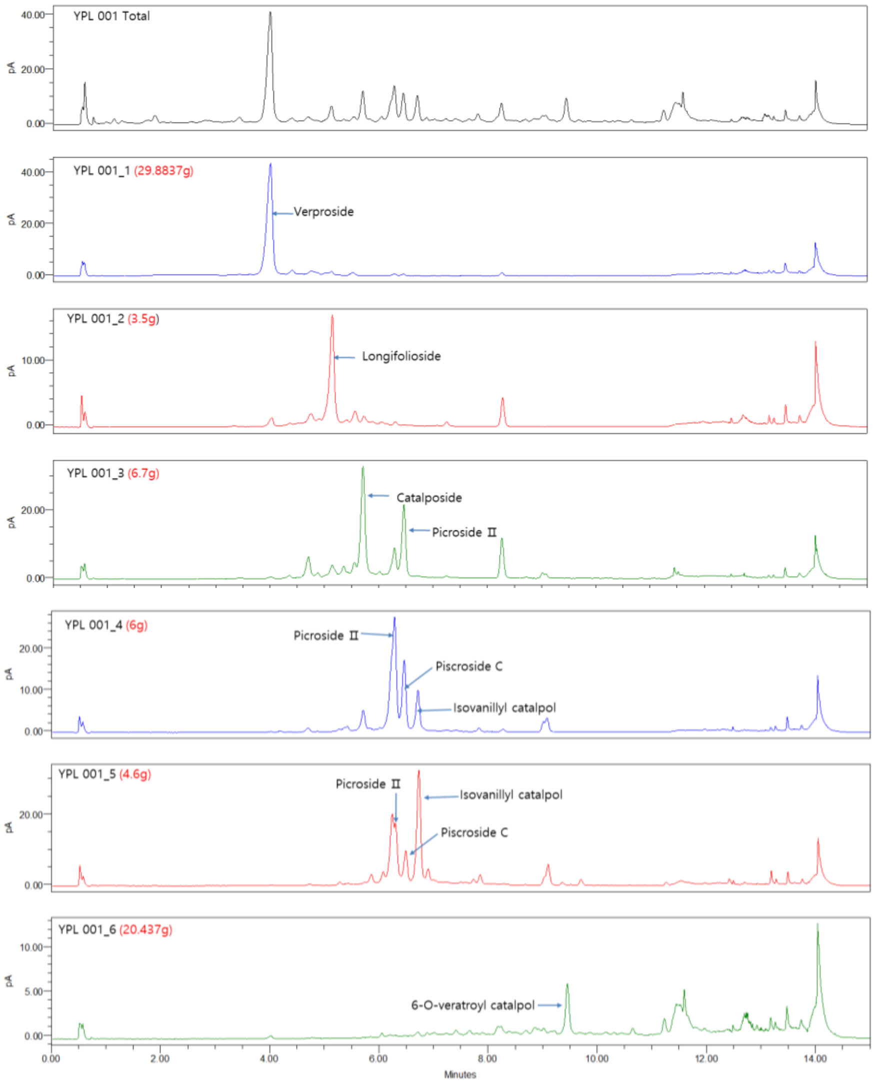

Figure S1-2. UPLC-PDA of fractions of *P. rotundum* var. *subintegrum* extract. YPL001_1: Fr.1 and Fr.2, YPL001_3: Fr.3, YPL001_4: Fr.4, YPL001_5: Fr.5, YPL001_6: Fr.6

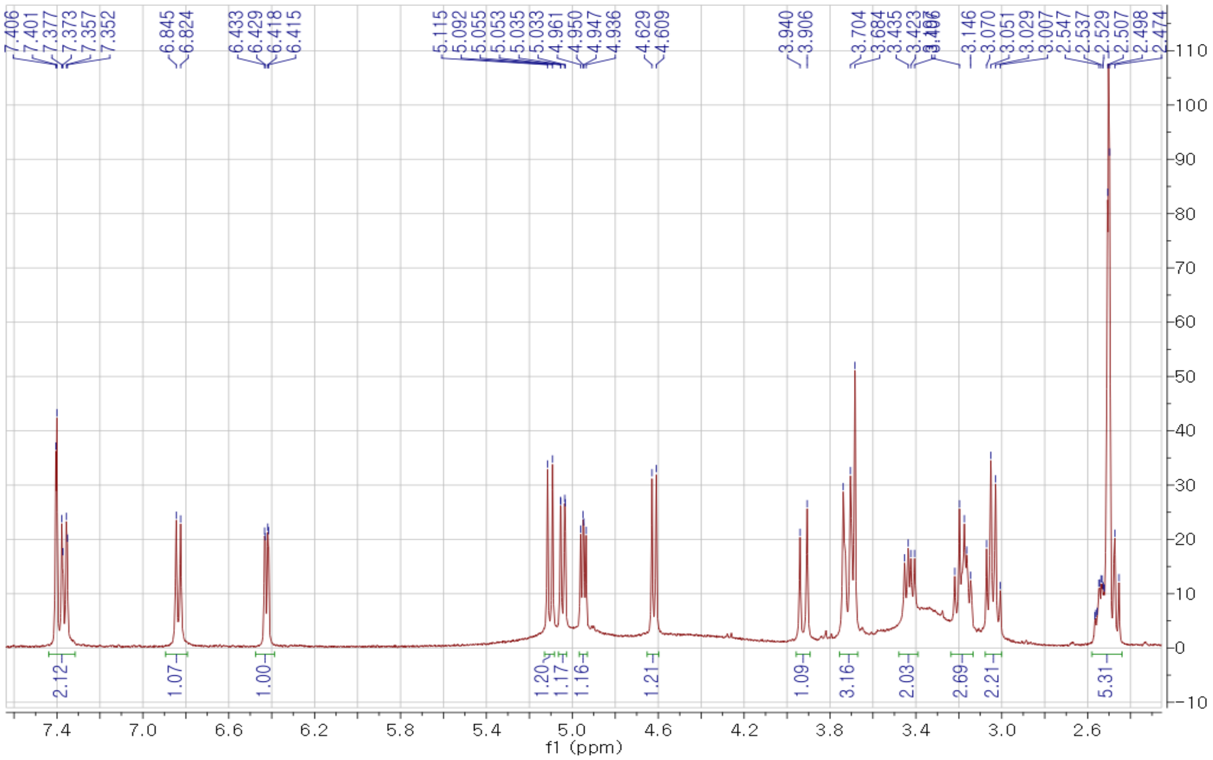

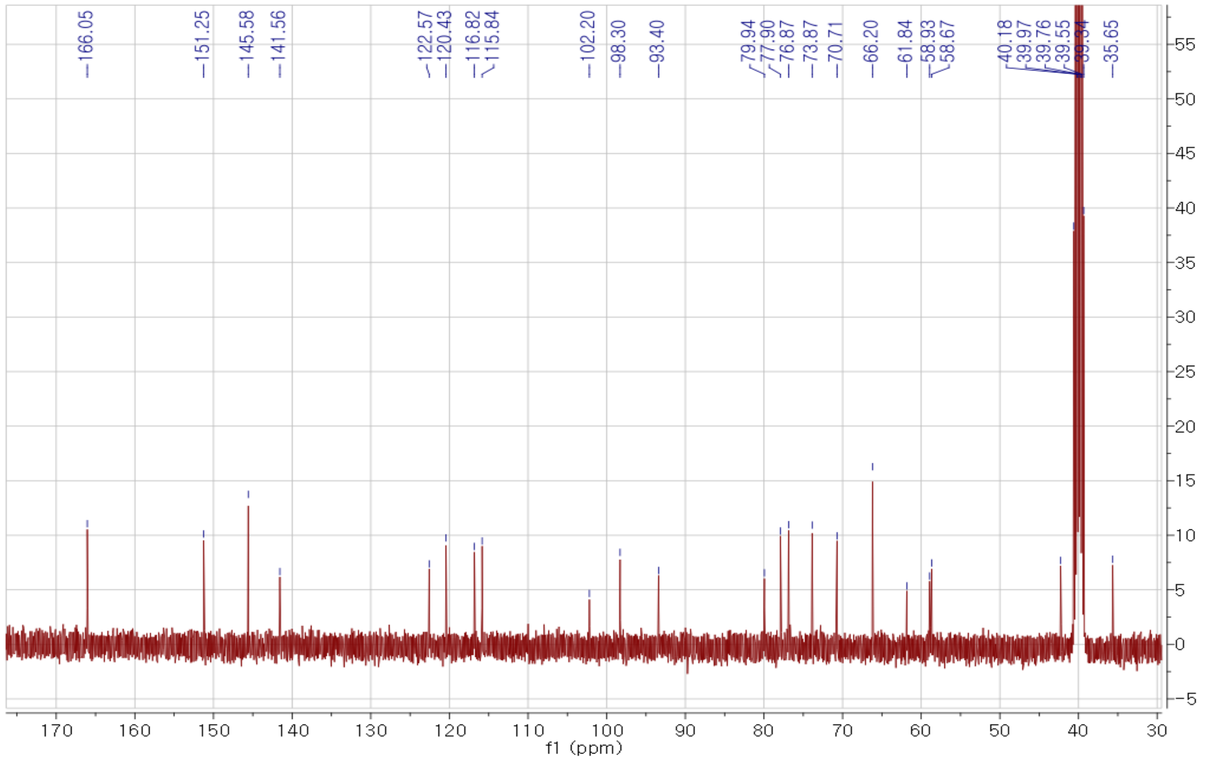

Figure S2-1. ^{1}H and ^{13}C NMR spectrum of Verproside (**1**).

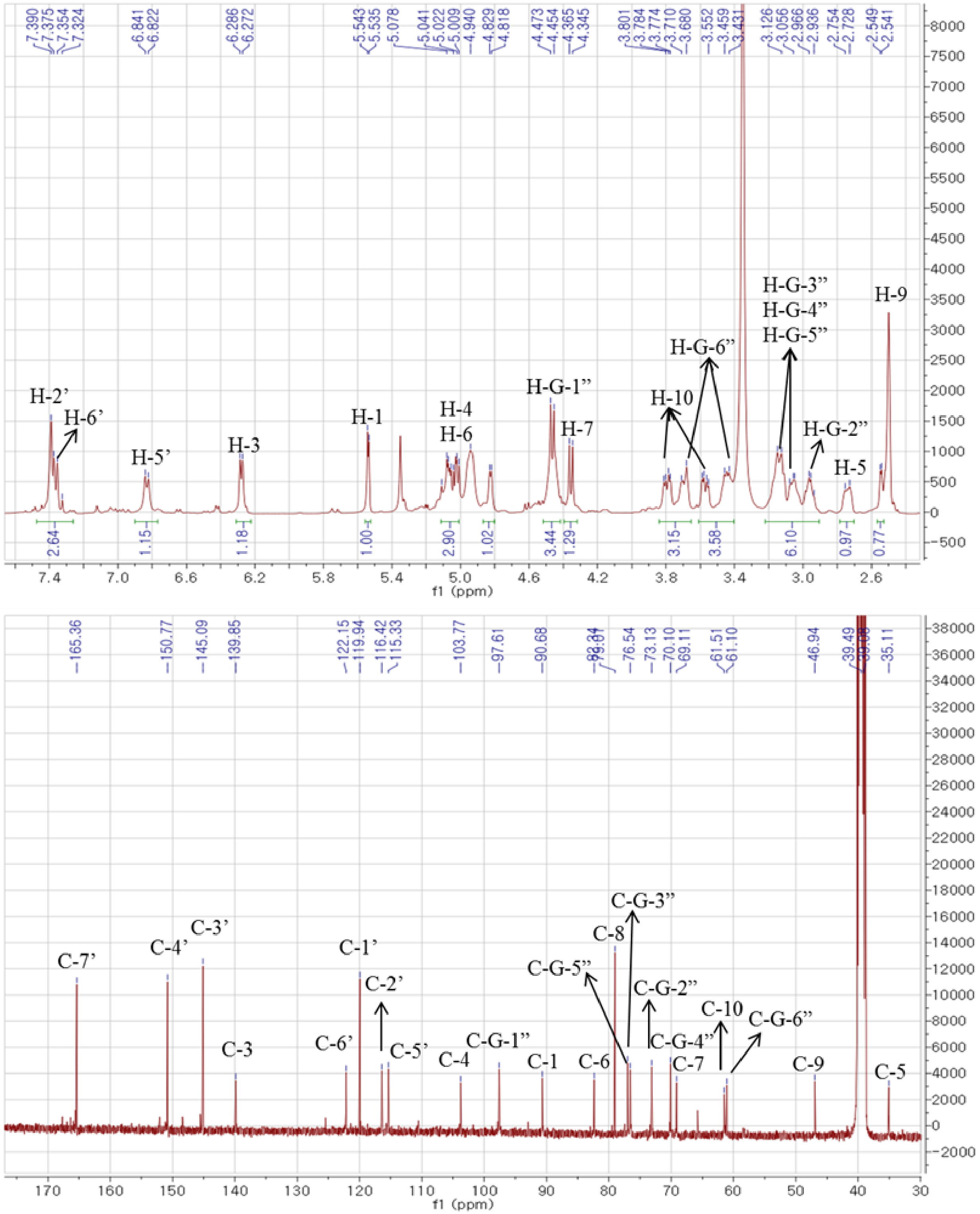

Figure S2-2. ^{1}H and ^{13}C NMR spectrum of Logifolioside A (**2**).

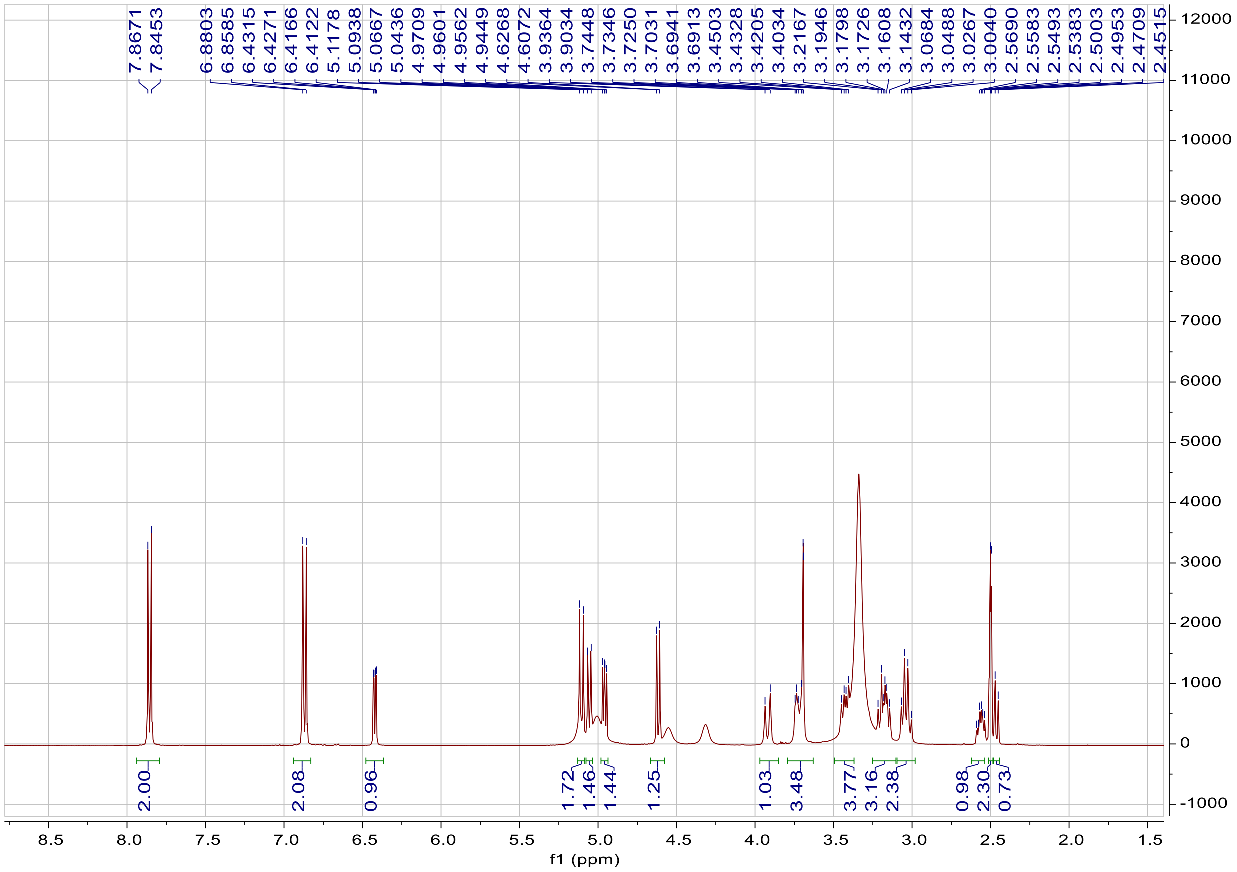

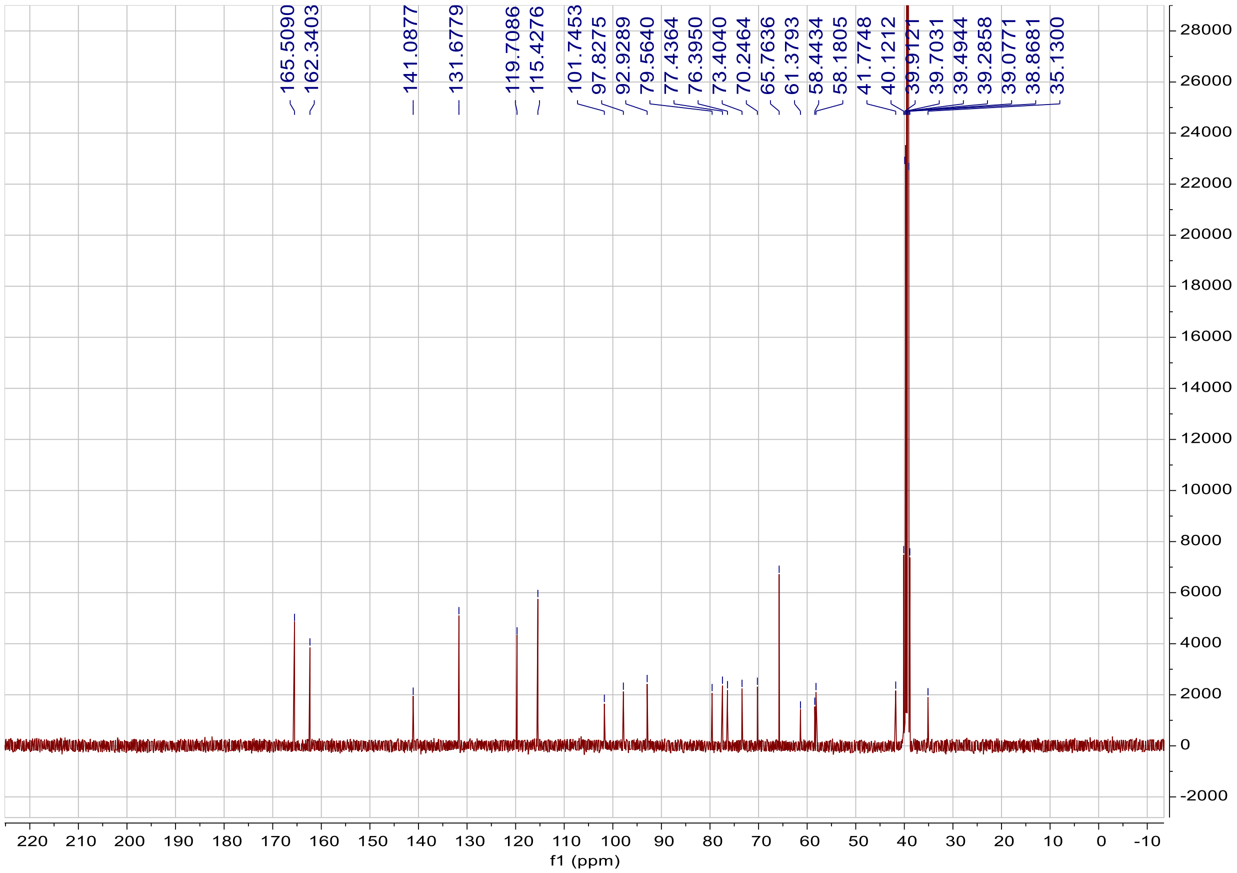

Figure S2-3. ^{1}H and ^{13}C NMR spectrum of Catalposide (**3**).

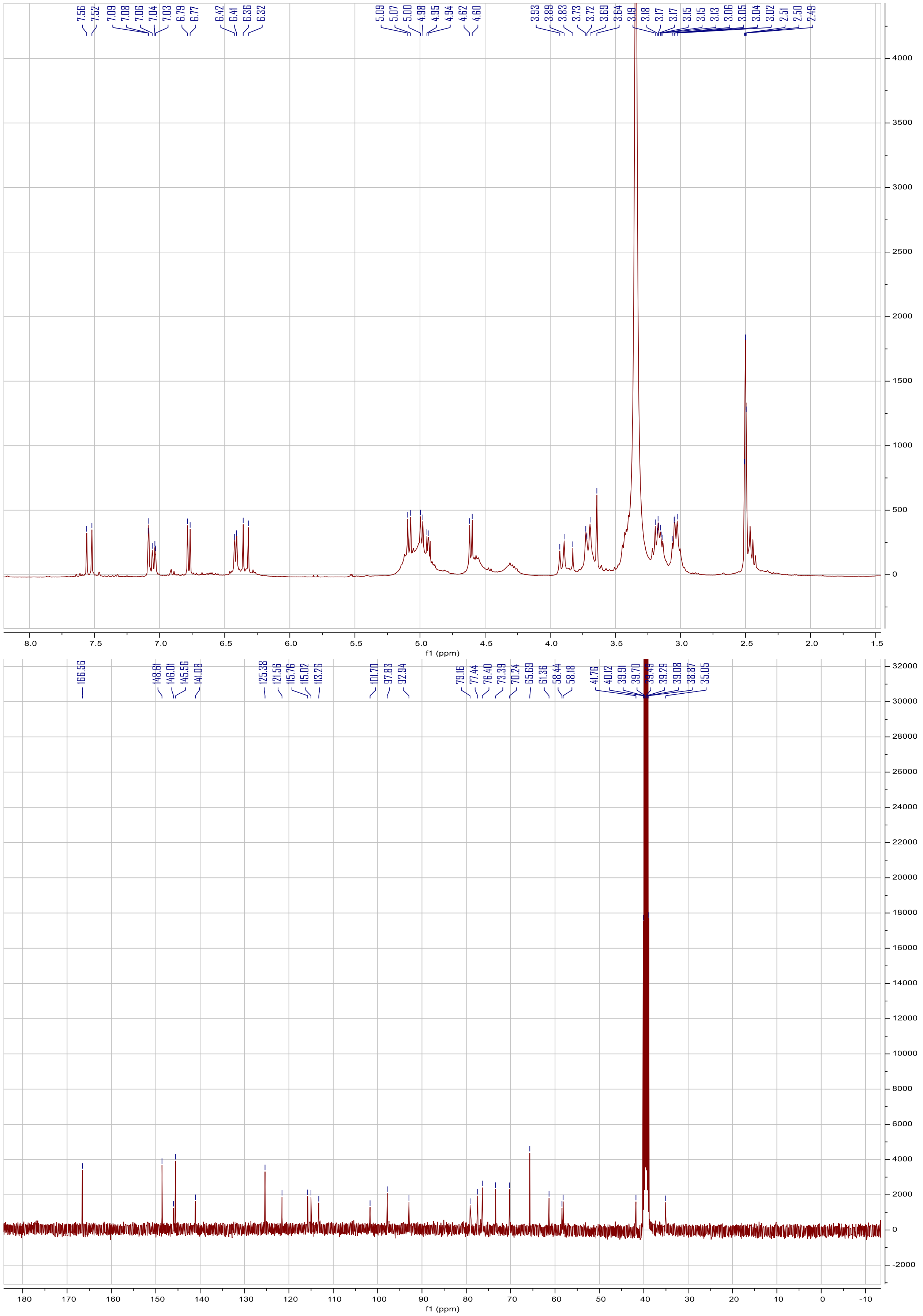

Figure S2-4. ^{1}H and ^{13}C NMR spectrum of Verminioside (**4**).

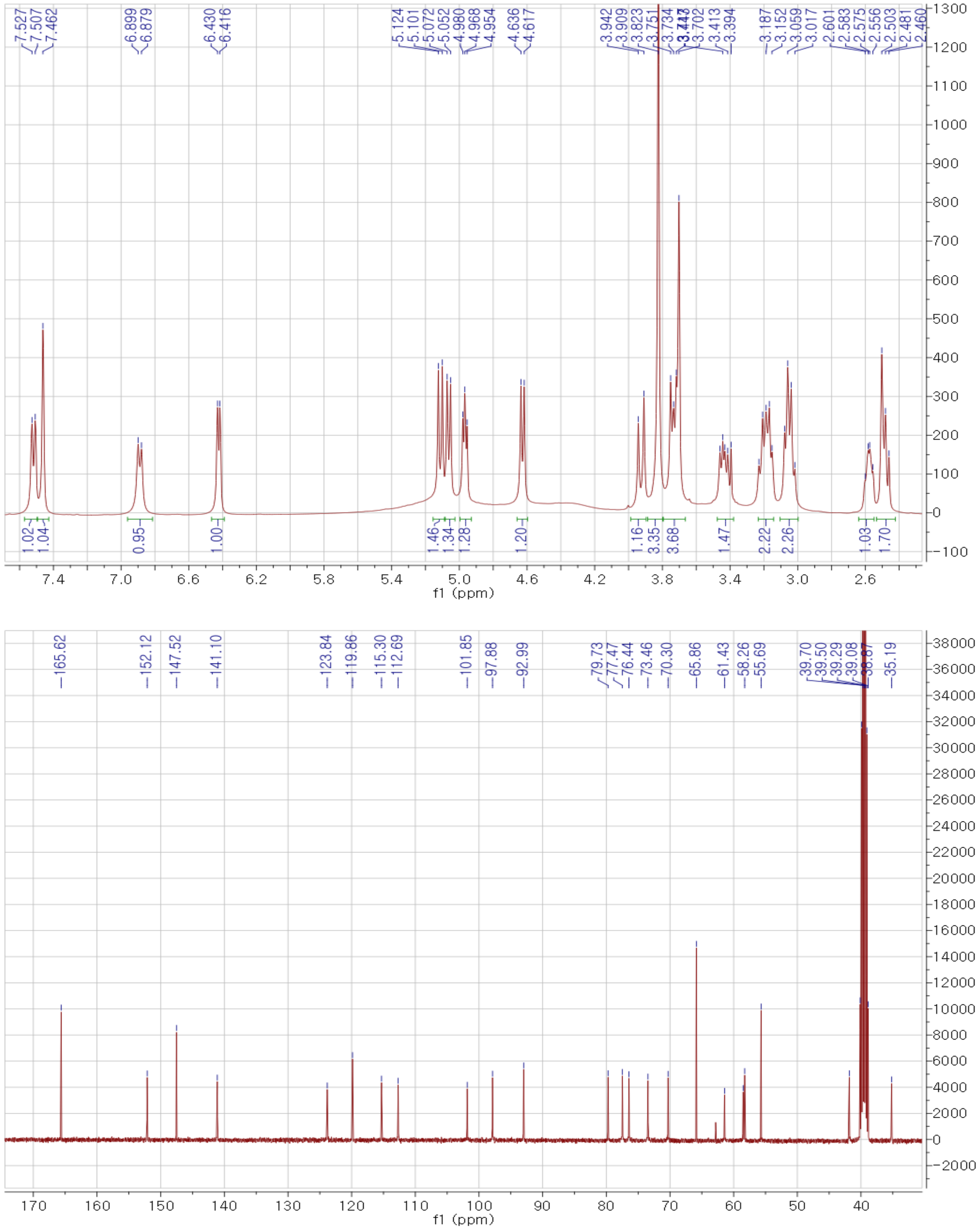

Figure S2-5. ^{1}H and ^{13}C NMR spectrum of Picroside II (**5**).

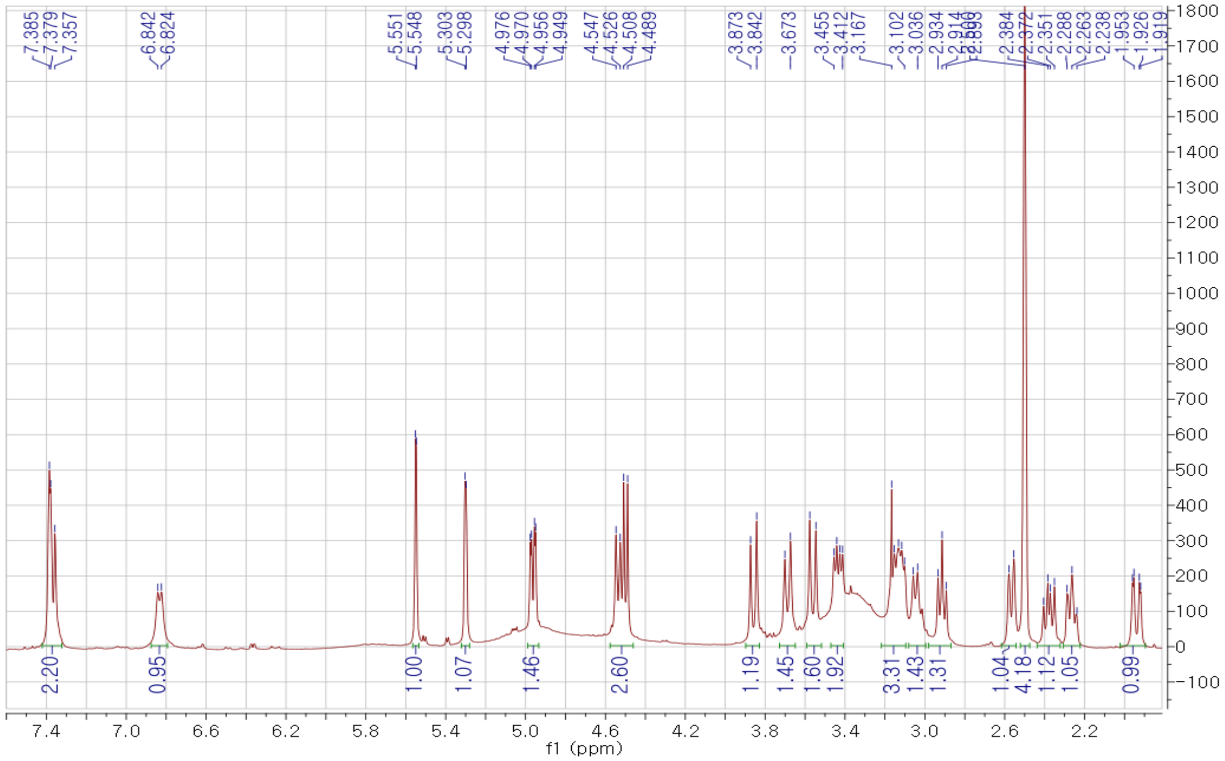

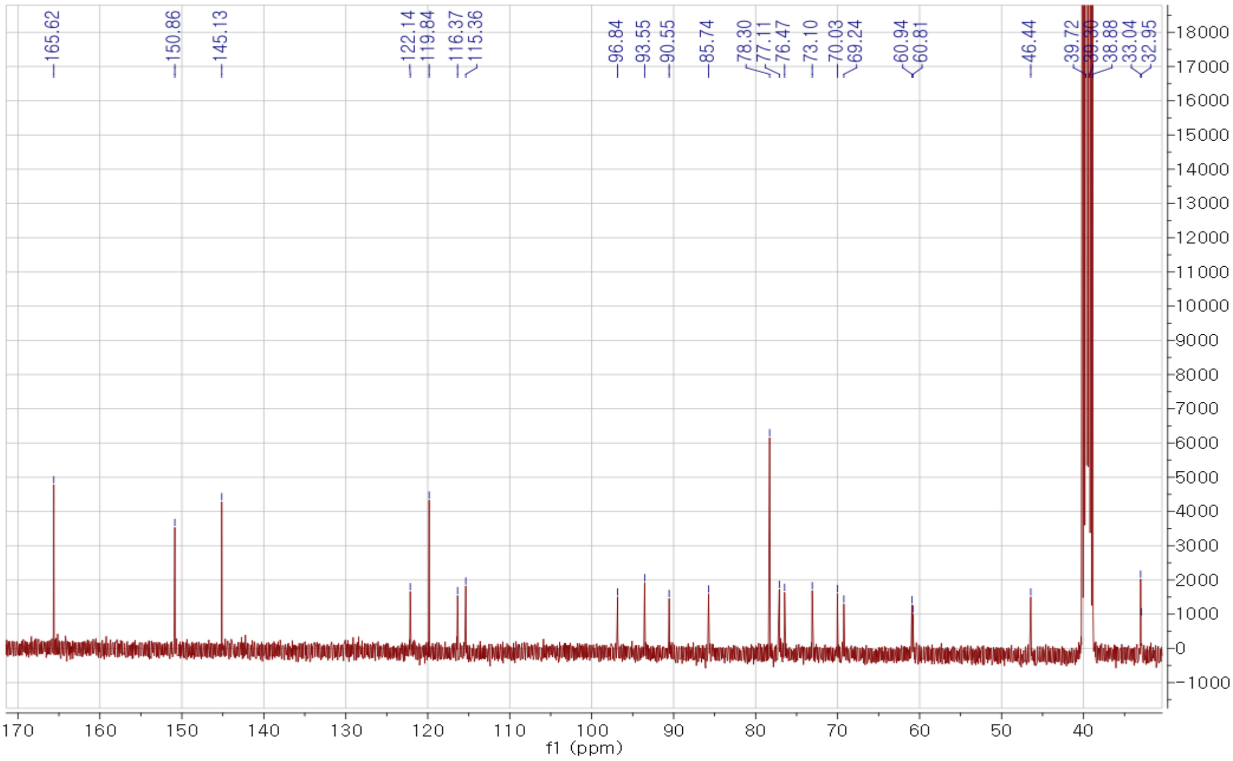

Figure S2-6. ^{1}H and ^{13}C NMR spectrum of Piscroside C (**6**).

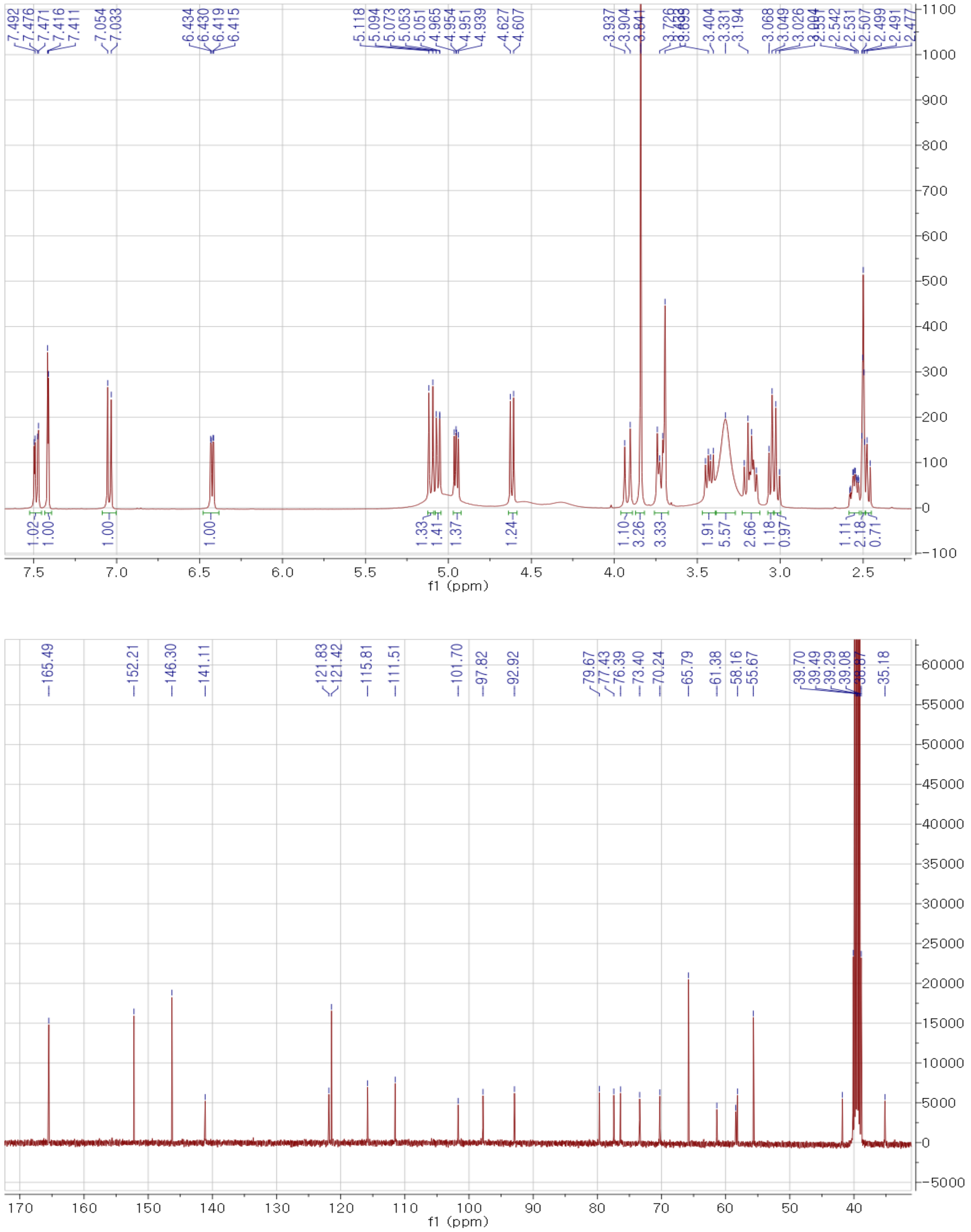

Figure S2-7. ^{1}H and ^{13}C NMR spectrum of Isovanillyl catalpol (**7**).

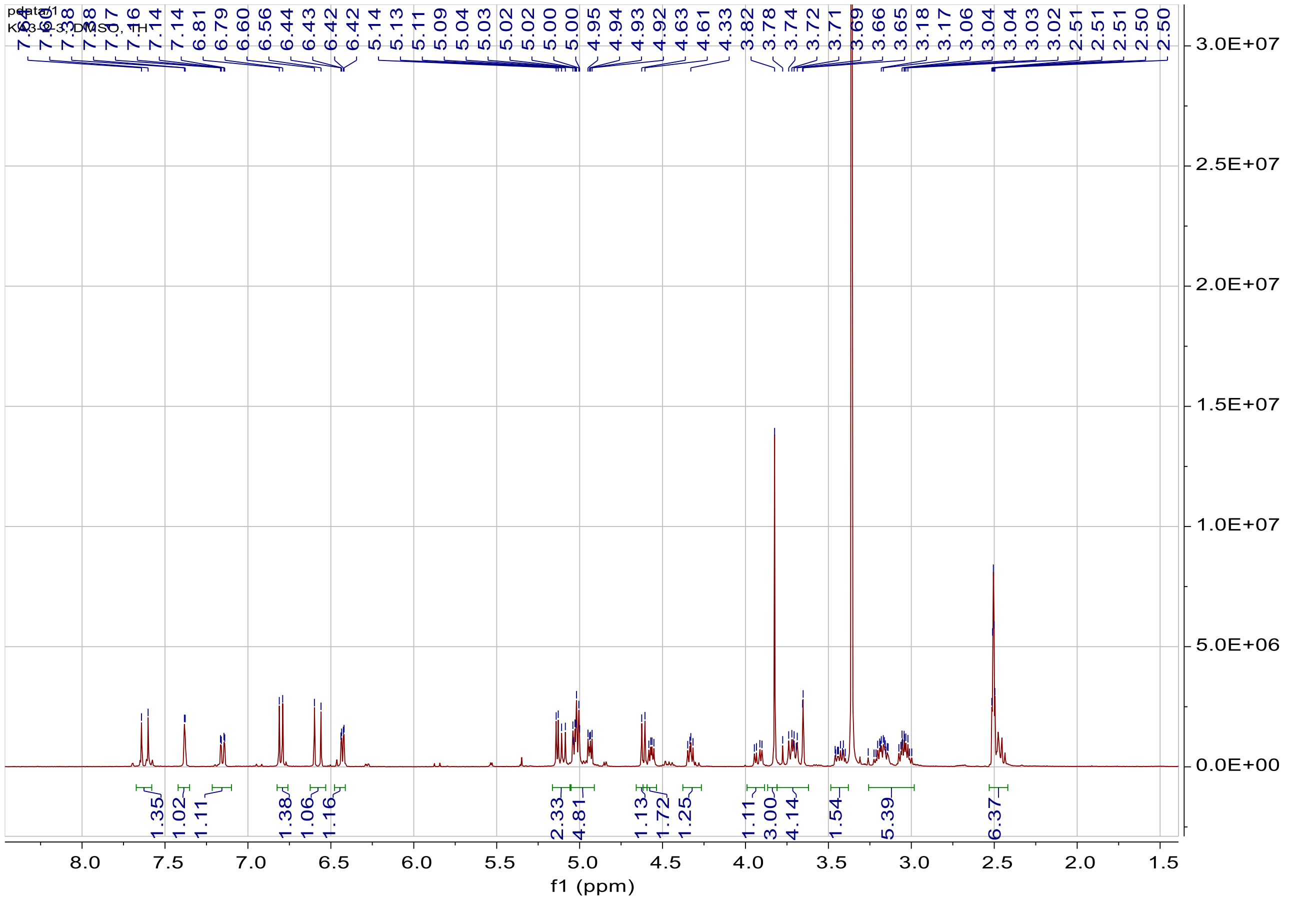

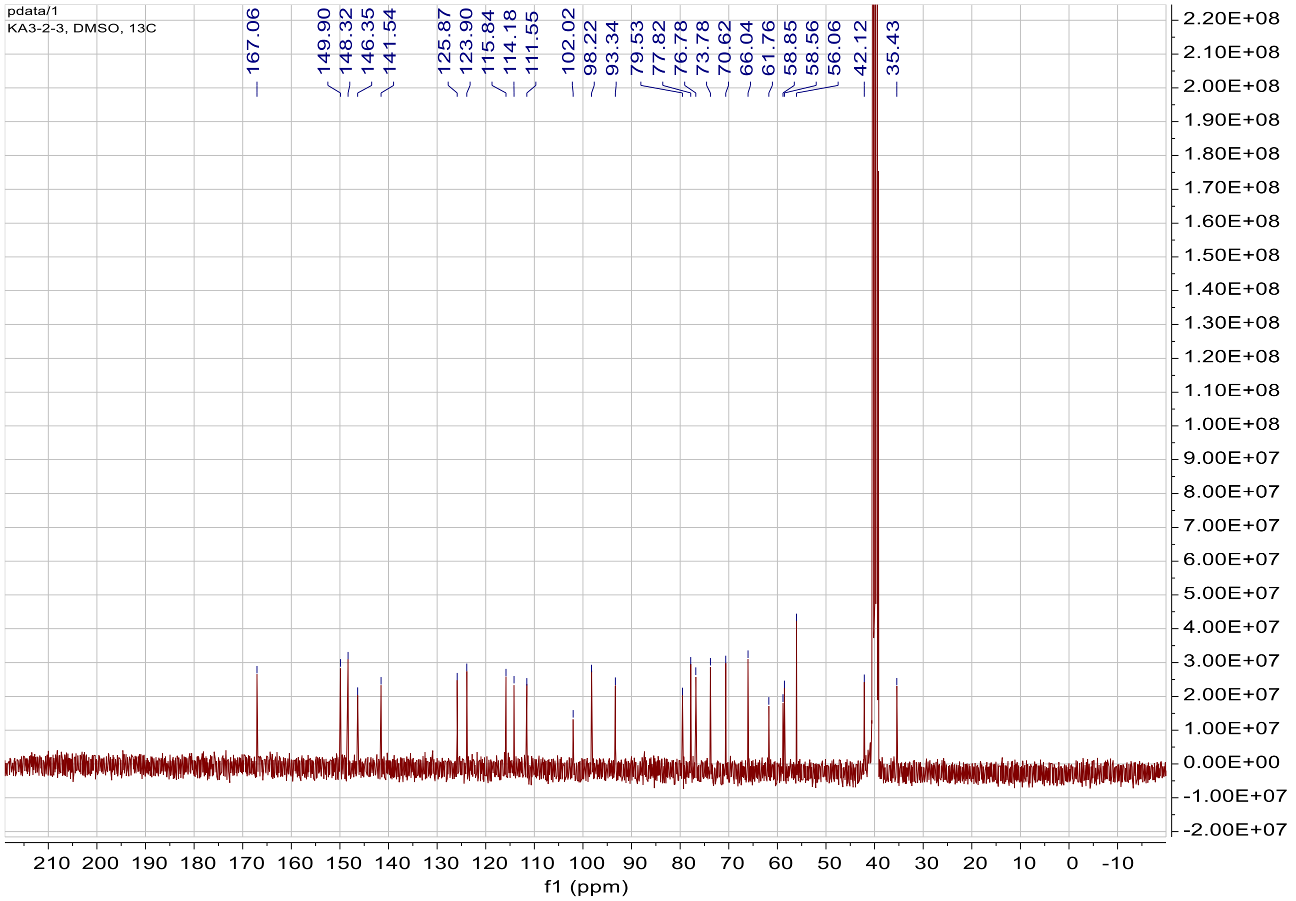

Figure S2-8. ^{1}H and ^{13}C NMR spectrum of Minecoside (**8**).

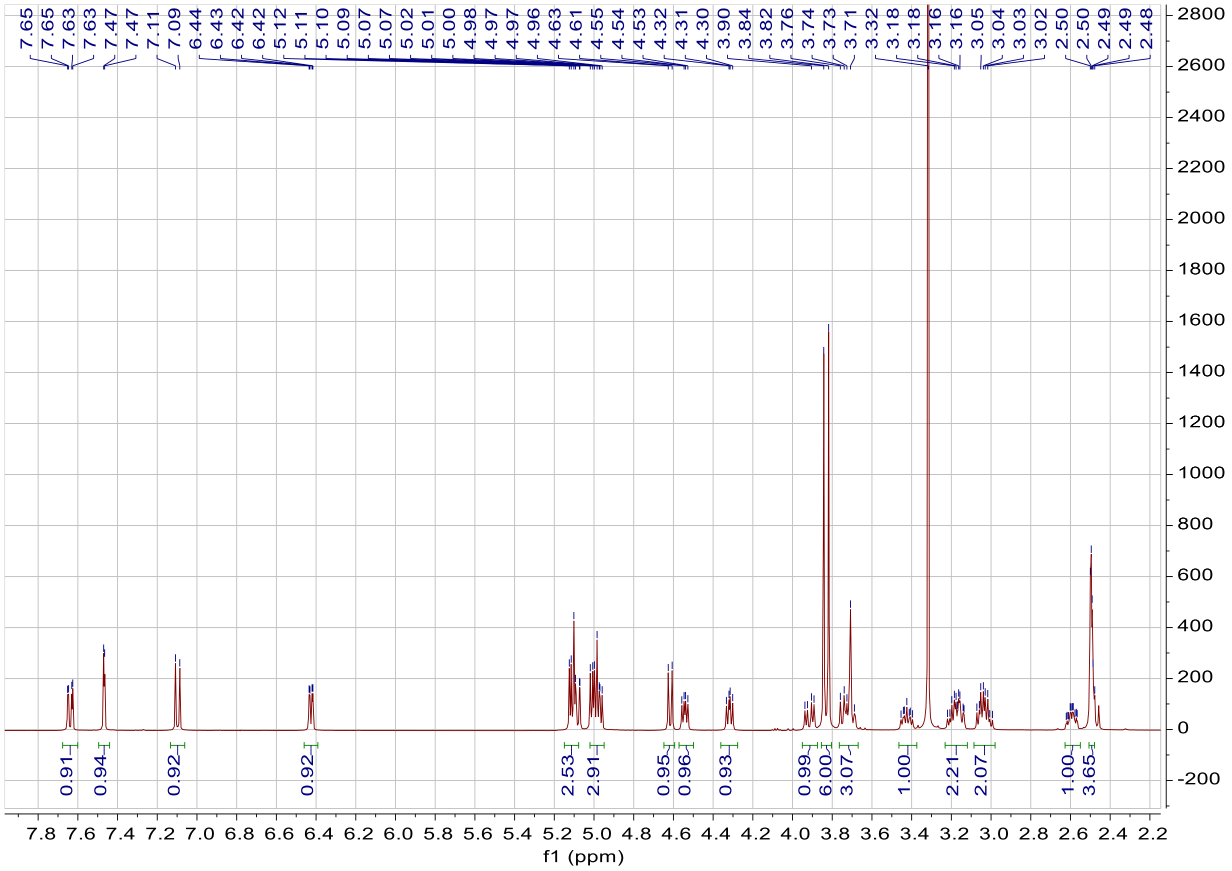

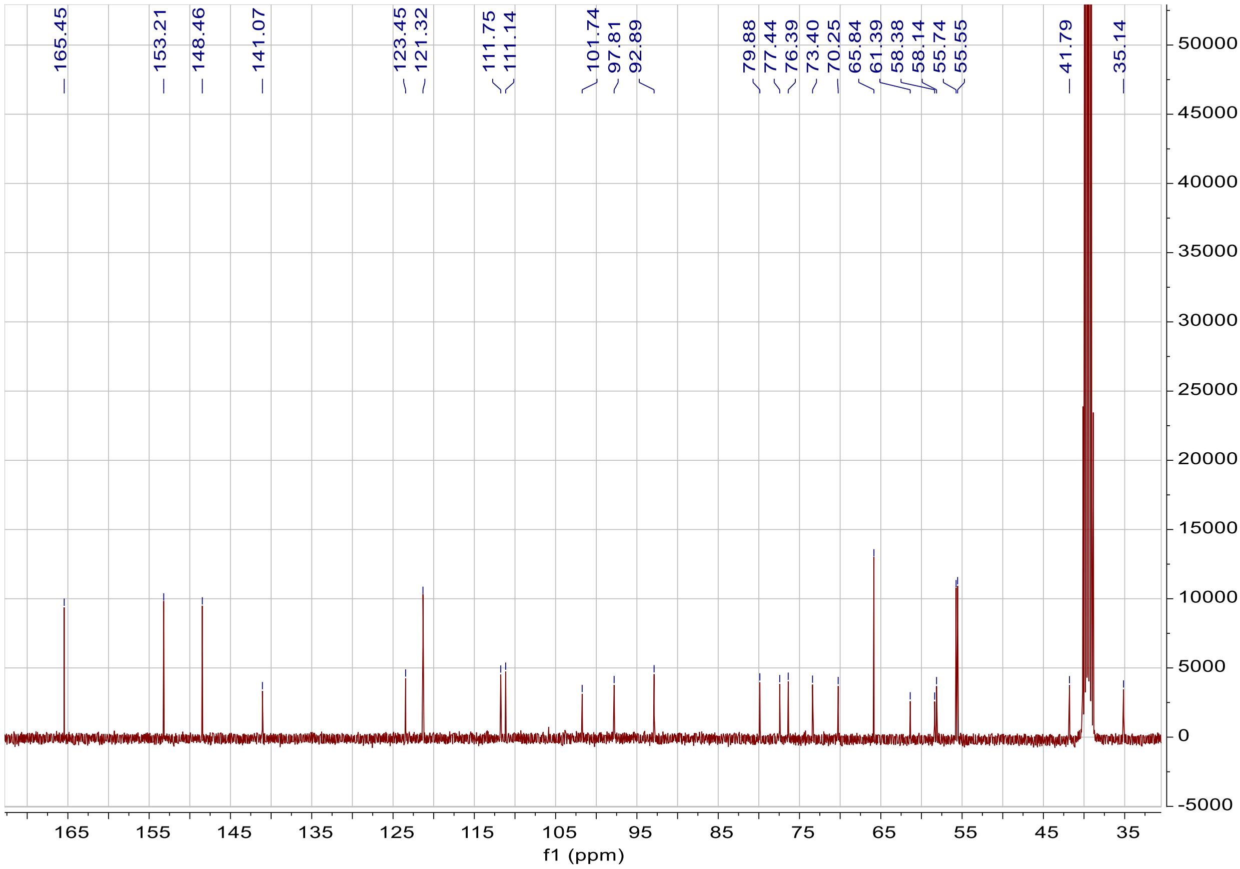

Figure S2-9. ^{1}H and ^{13}C NMR spectrum of 6-*O*-Veratroyl catalpol (**9**).

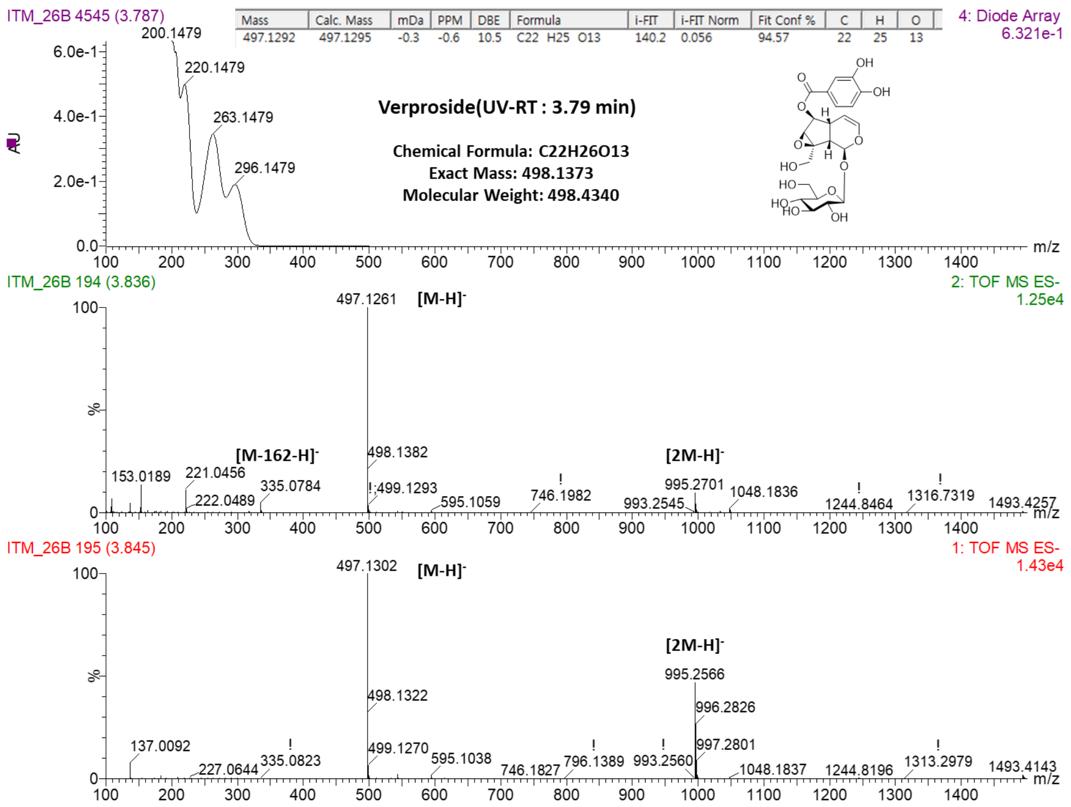

Figure S3-1. UV, MS/MS and MS data of Verproside (**1**).

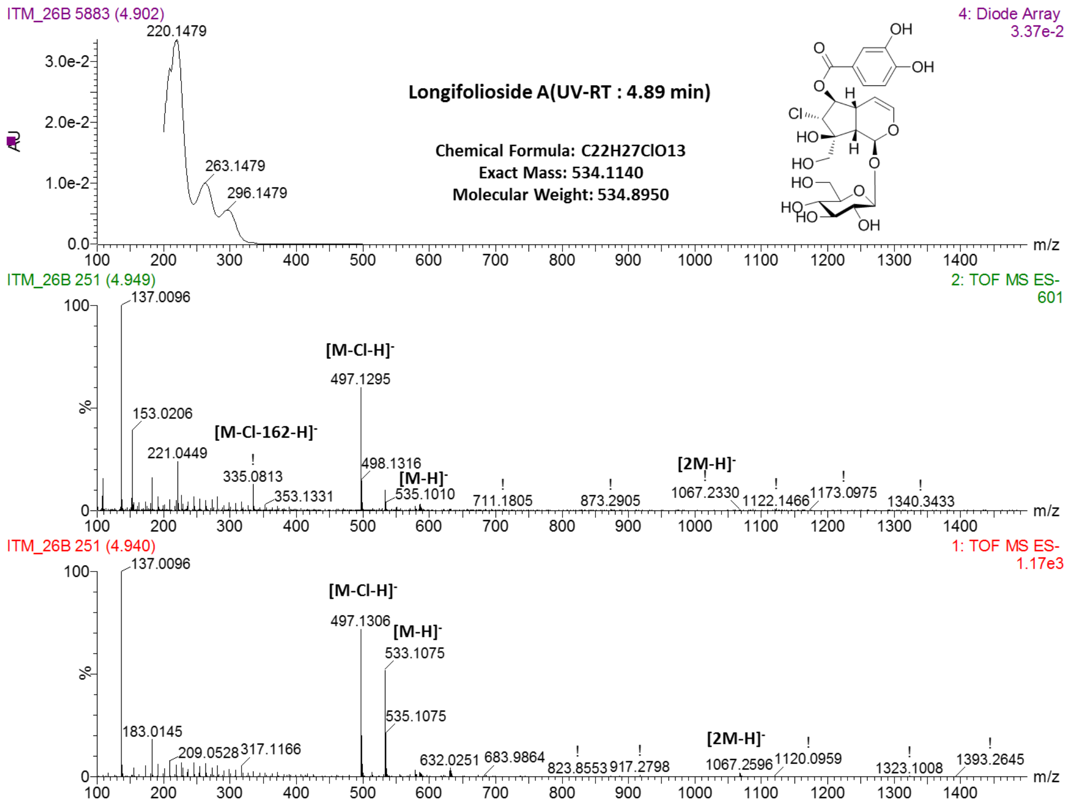

Figure S3-2. UV, MS/MS and MS data of Longifolioside A (**2**).

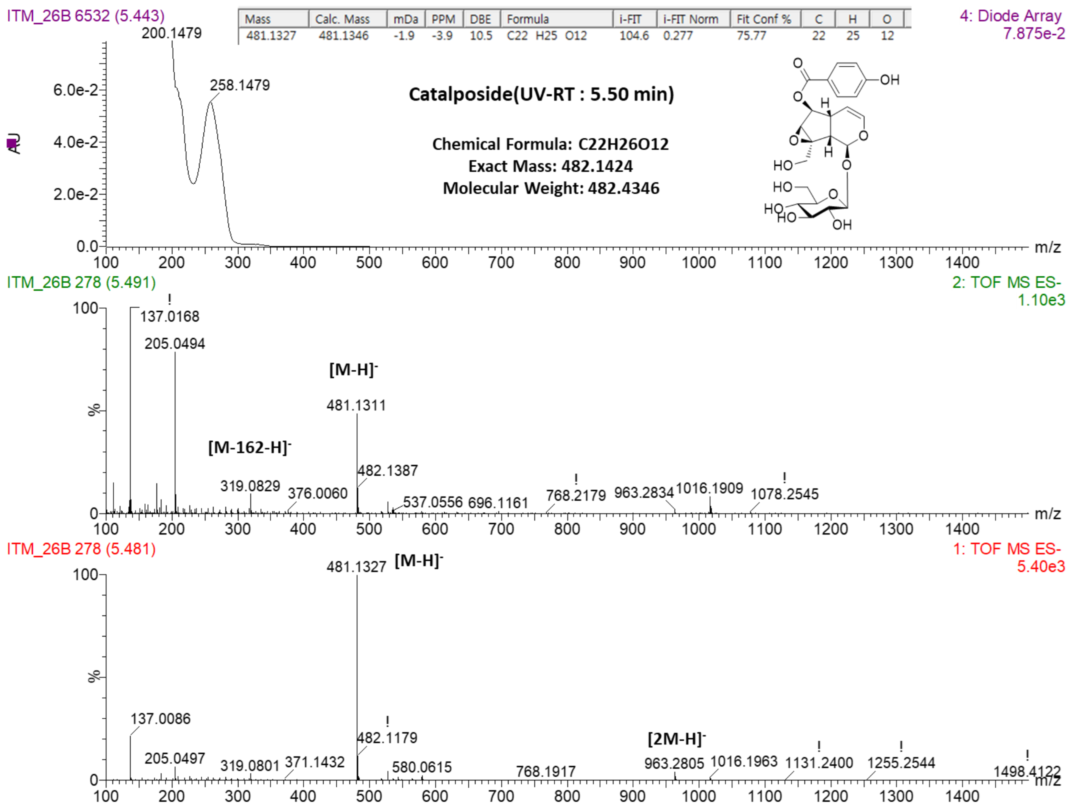

Figure S3-3. UV, MS/MS and MS data of Catalposide (**3**).

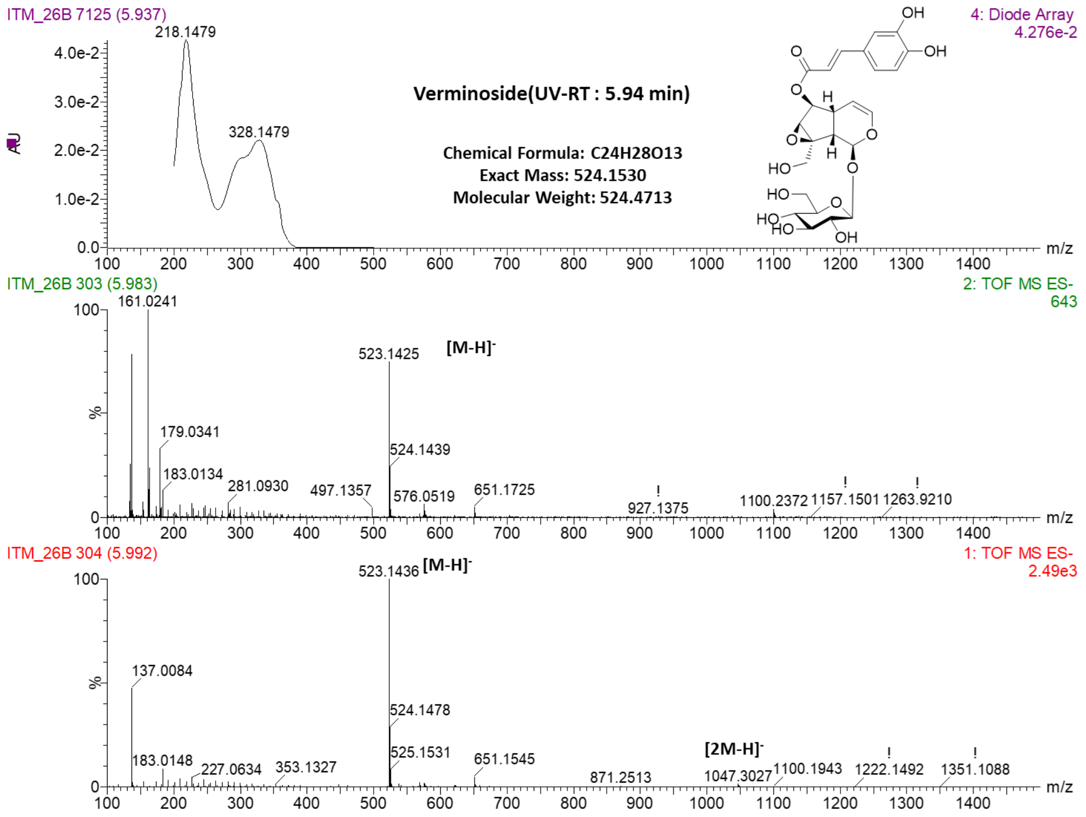

Figure S3-4. UV, MS/MS and MS data of Verminoside (**4**).

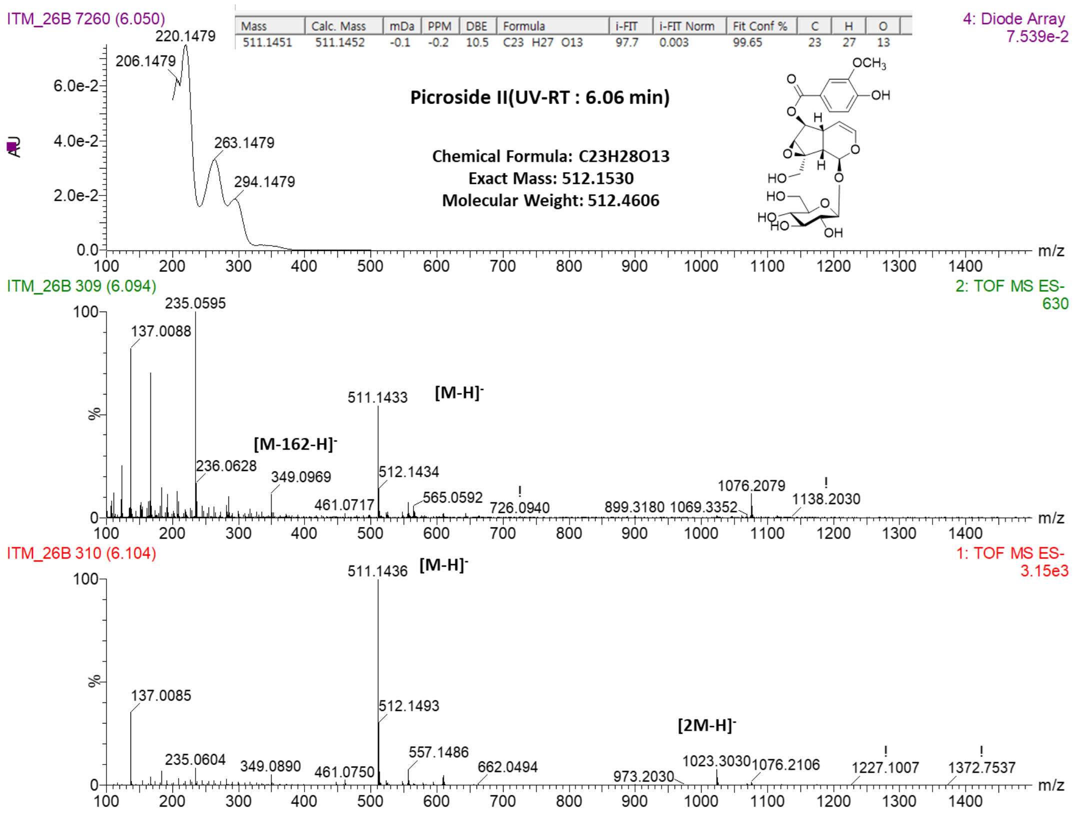

Figure S3-5. UV, MS/MS and MS data of Picroside II (**5**).

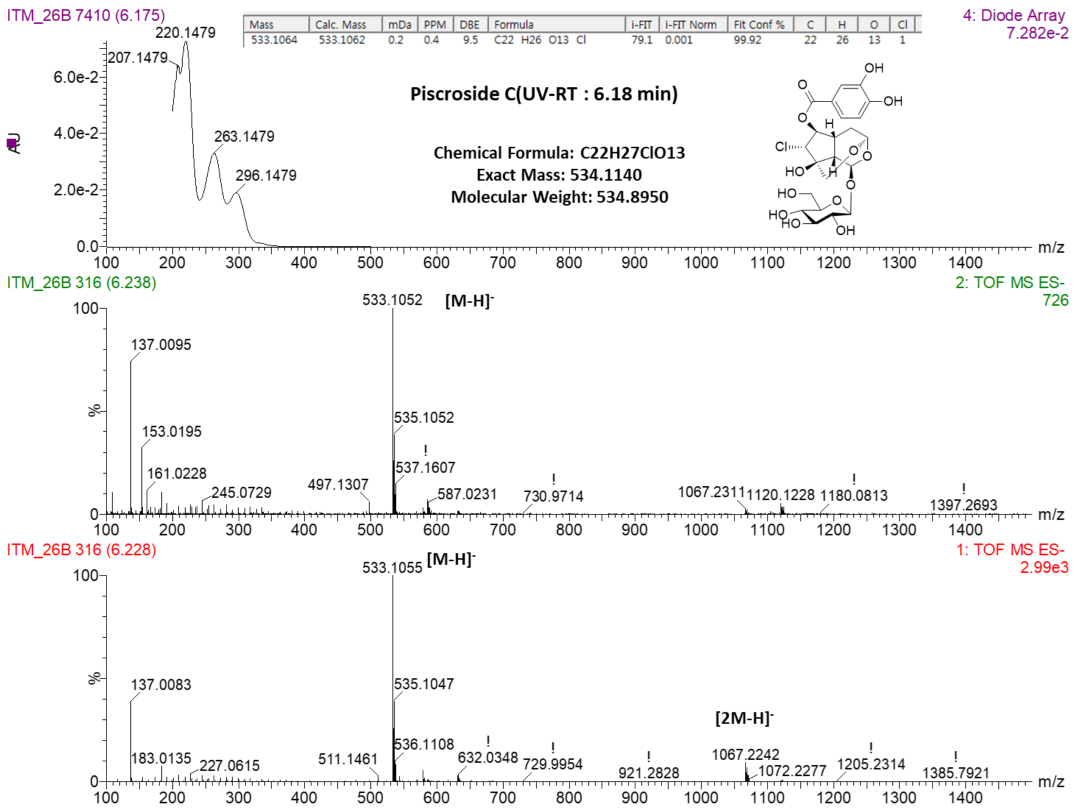

Figure S3-6. UV, MS/MS and MS data of Piscroside C (**6**).

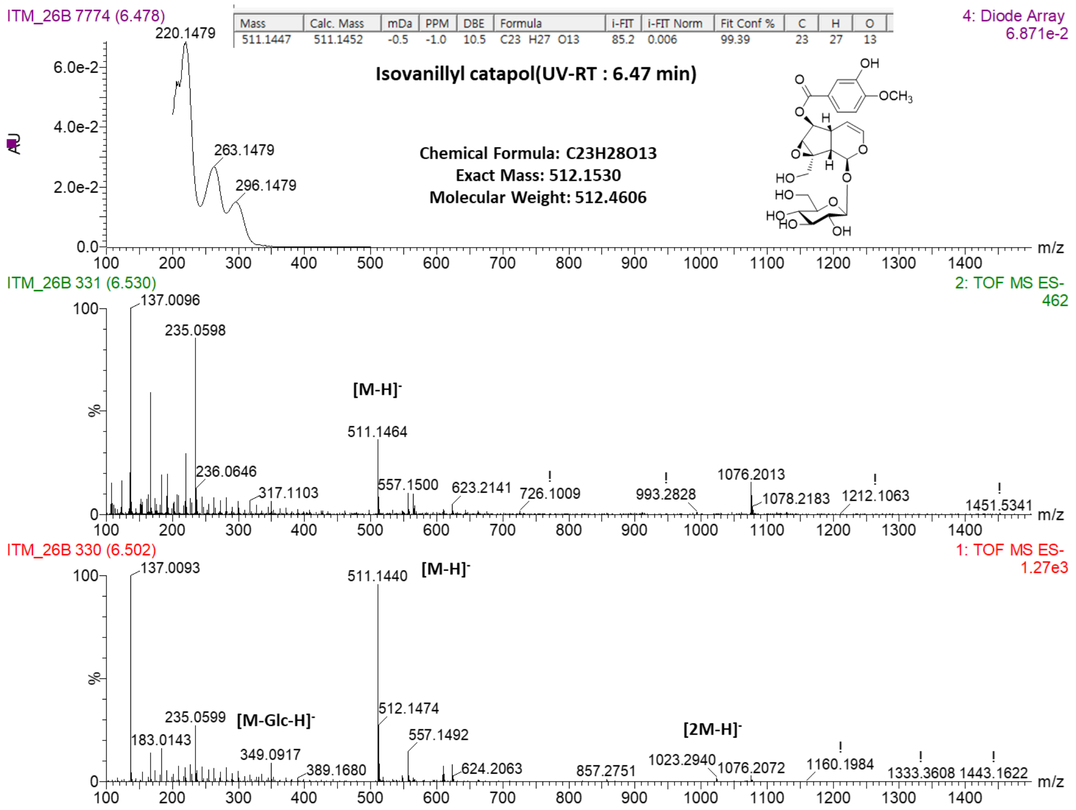

Mass	Calc. Mass	mDa	PPM	DBE	Formula	i-FIT	i-FIT Norm	Fit Conf %	C	H	O
511.1447	511.1452	-0.5	-1.0	10.5	C23 H27 O13	85.2	0.006	99.39	23	27	13

Figure S3-7. UV, MS/MS and MS data of Isovanillyl catalpol (**7**).

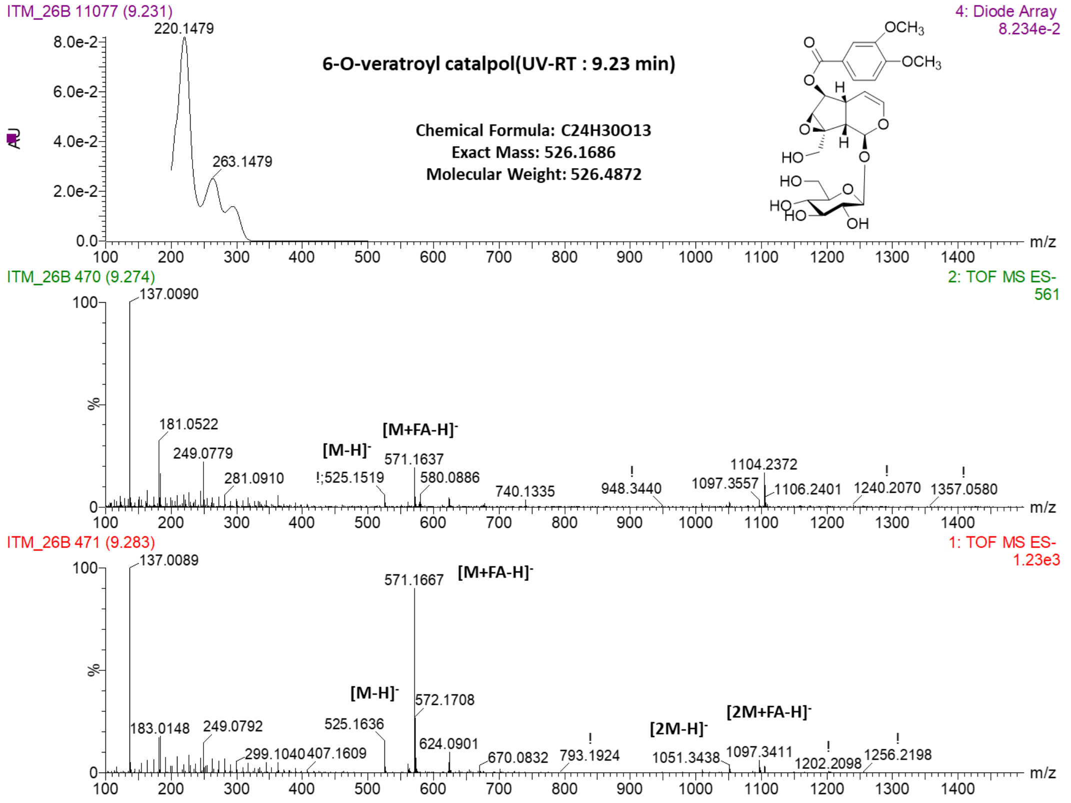

Figure S3-8. UV, MS/MS and MS data of Minecoside (**8**).

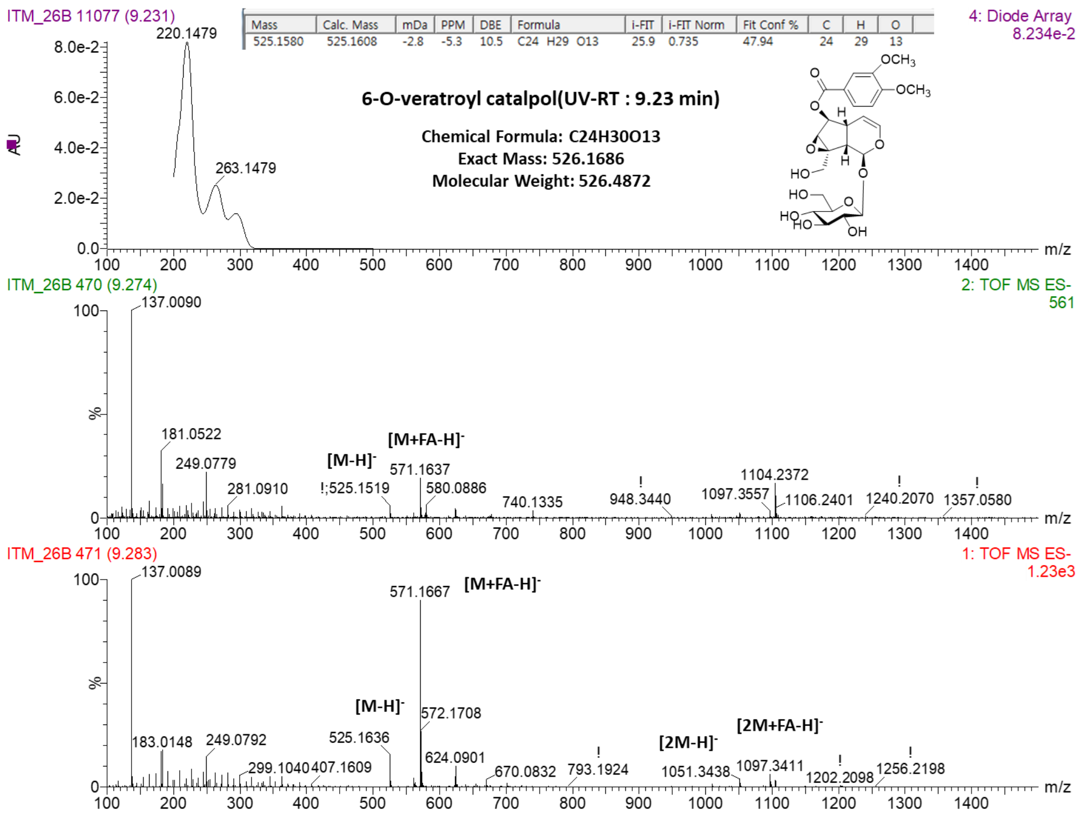

Figure S3-9. UV, MS/MS and MS data of 6-*O*-Veratroyl catalpol (**9**).

Cell maintenance

The B16F10 melanoma and Melan-A normal murine melanocyte cells were purchased from the American Type Culture Collection (ATCC), respectively. The B16F10 cells were cultured in complete Dulbecco's Modified Eagle's Medium (DMEM high glucose, Gibco) supplemented with 10% (v/v) Fetal Bovine Serum (FBS, Gibco, USA), 100 U/mL penicillin, 50 μg/ml streptomycin. The Melan-A cells were cultured in RPMI-1640 medium supplemented with 10% FBS, 2 mM L-glutamine, 200 nM 12-o-tetradecanoyl phorbol-13-acetate (Sigma, USA), 100 U/mL penicillin, 50 μg/ml streptomycin, and grown in a humidified atmosphere containing 5% CO_2 in air at 37°C.

Cell viability assay

The cells were seeded in 96-well plates at a density of 5×10^3 cells/well in cultured medium. The cells were treated with the corresponding concentrations of verproside for 48 h. Cell viability was measured in triplicate using a Cell Counting Kit-8 (CCK-8; Dojindo Molecular Technologies, Rockville, MD, USA) . Absorbance was determined using an Epoch microplate reader (BioTek Instruments, Winooski, VT, USA, #CK04) and calculated as a relative percentage (%) of the control value.

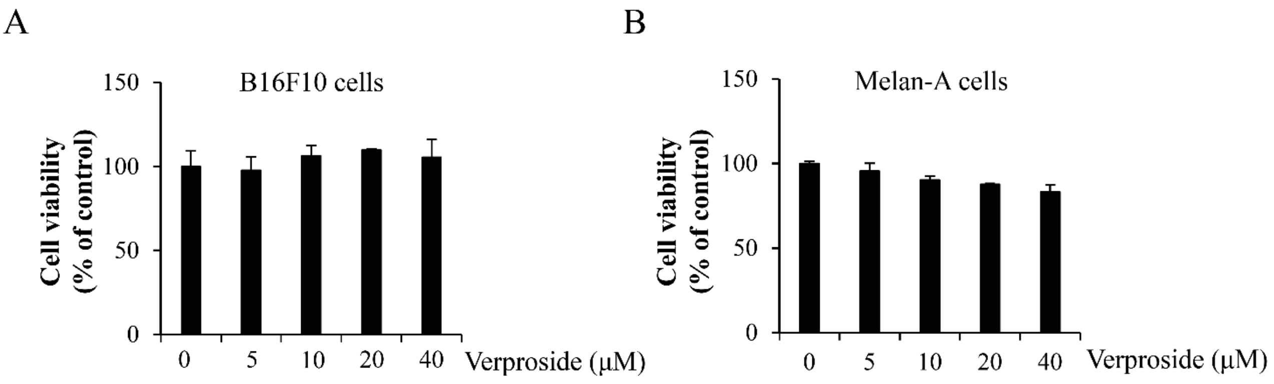

Figure S4. The cell viability by verproside in B16F10 and Melna-A cells. (A) CCK-8 assays in B16F10 cells. (B) CCK-8 assays in Melna-A cells.

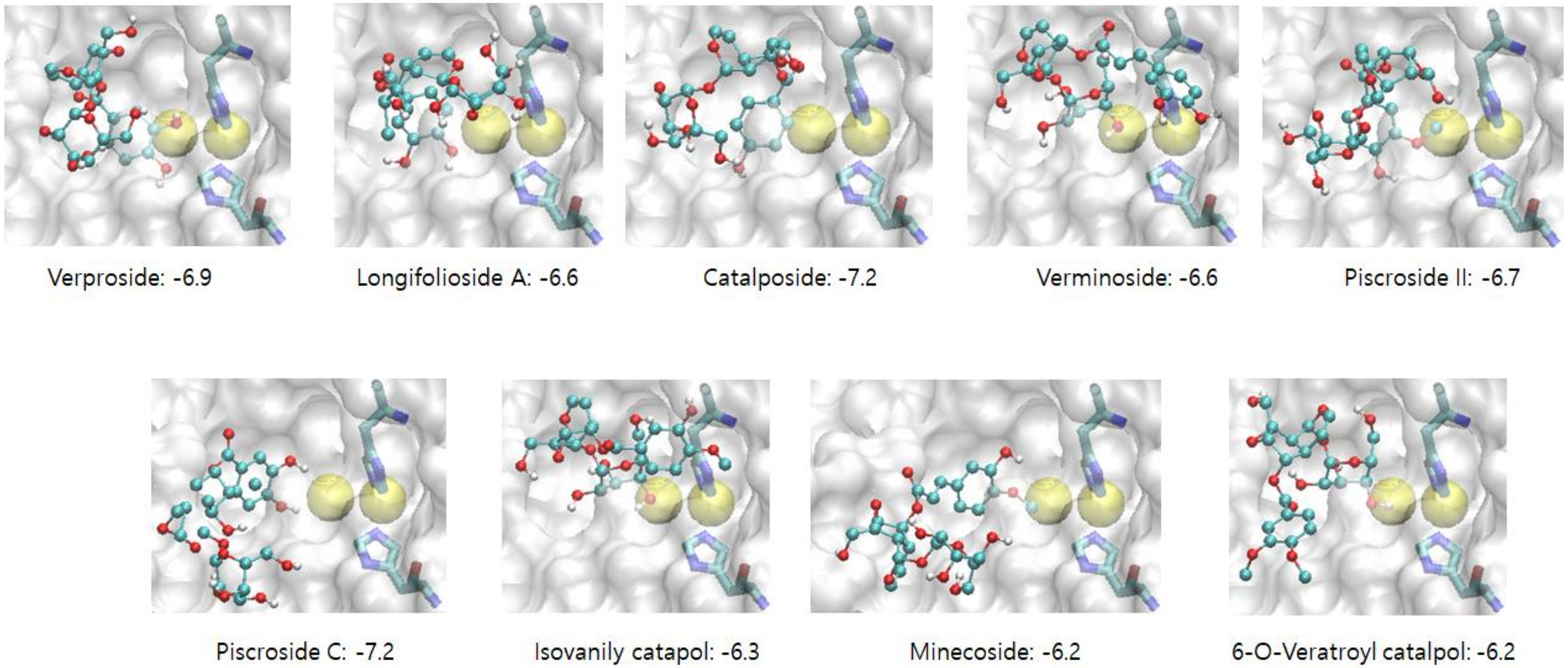

Figure S5. Structural views of nine compounds on the active site of mushroom tyrosinase. The numbers next to the compound names are binding energy in kcal/mol. Tyrosinase is drawn by white surface protein model, and two Copper ions existing on tyrsoinase is represented by yellow space-fill model. Each compound is drawn by ball and stick model and colored by red (Oxygen), cyan (Carbon), and white (Hydrogen). The interacting residues on tyrosinase is two, HIS61 and HIS85, drawn by stick models colored by blue (Nitrogen).

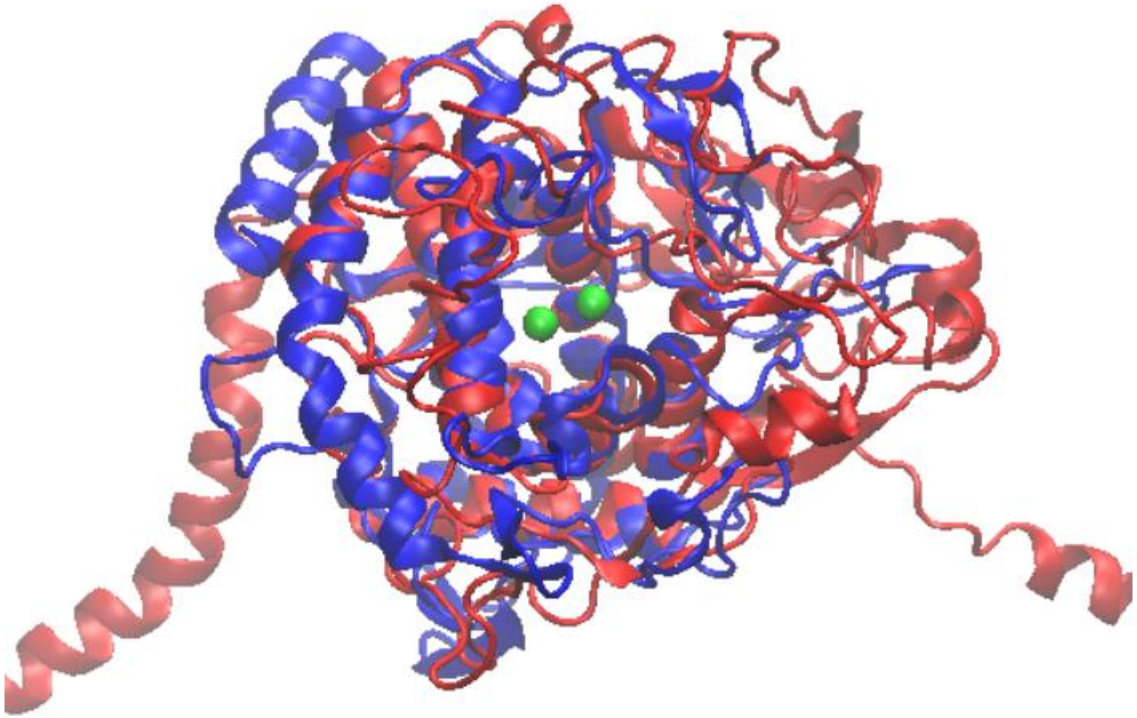

Figure S6. Structural comparisons on mushroom (mTyr) and human tyrosinases (hTyr). The structures are drawn by cartoon molecular views colored by blue (mTyr) and red (hTyr). Two Copper ions are drawn by green spacefill models.

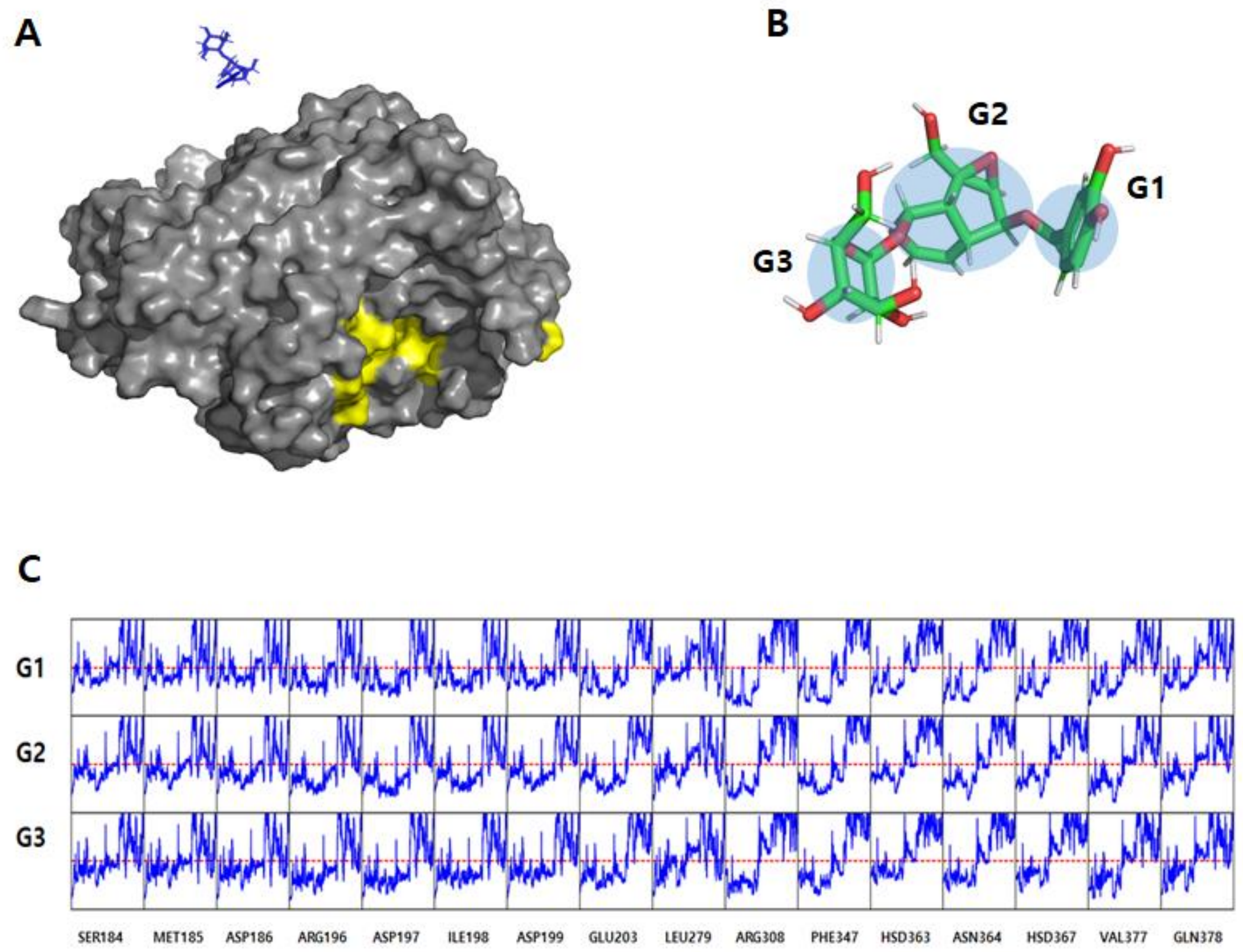

Figure S7. Molecular dynamics simulation results. (A) The final structures of hTyr-verproside complex. The tyrosinases are depicted using a grey surface model, while the active site revealed by docking simulation is colored yellow. The verproside is represented by a blue stick model. For clarity, the explicit water models and counter ions are not included in the figure. (B) Verproside grouping. The center of geometry (COG) of the atoms comprising the rings was utilized to measure the interacting distance. The measurement was conducted between the COG and the alpha Carbon atom of the interacting residues. (C) The interaction distance between verproside and mTyr. It consists of 48 sub-panels, each displaying the distance-time profile (X-axis: time from 0 to 100 ns; Y-axis: distance from 0 to 20 Å). The Y panel is divided into three groups, namely G1, G2, and G3, as mentioned in Figure 6B. The red dotted line represents the distance of 10 Å, which serves as the lower limit distance.

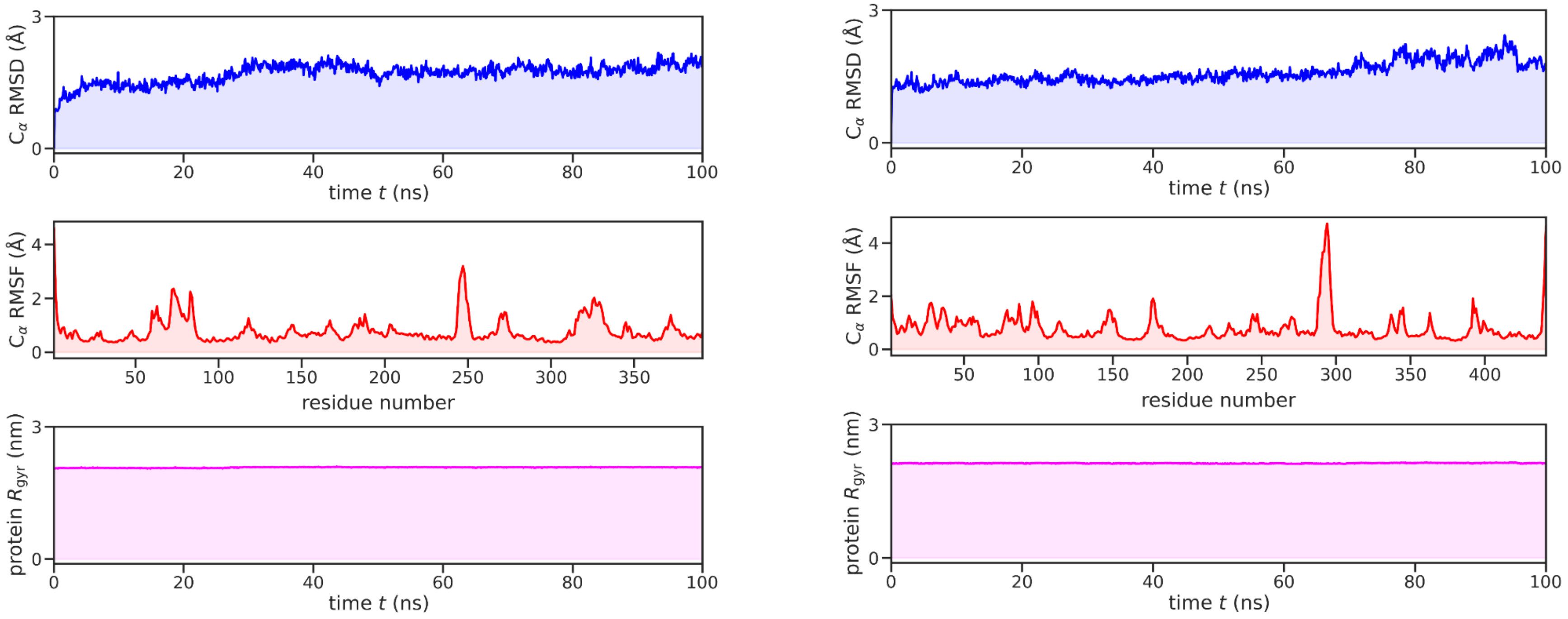

Figure S8. The structure validation during MD simulation for mTyr (left) and hTyr (right). Each panel has three sub panels, RMSD, RMSF, and Rgyr properties.

Table S1. The method conditions of ultrafiltration samples.

Sample	Extract in buffer (10,000 ppm)	Tyrosinase (144 U/mL)	0.25M phosphate buffer (pH 6.8)	37 °C Incubate Time	Spin down	Total		
						Tyrosinase (U/mL)	Ext. (ppm)	Volume (μL)
1	5 mg in MeOH	-	-	-	13,000 rpm 20 min	-	1,000	500
2	50 μL	-	450 μL	-	13,000 rpm 20 min (Amicogen)	-	1,000	500
3	50 μL	50 μL	400 μL	20 min	13,000 rpm 20 min (Amicogen)	14.4 U	1,000	500
4	50 μL	50 μL	400 μL	15 min	13,000 rpm 20 min (Amicogen)	14.4 U	1,000	500
5	50 μL	100 μL	350 μL	15 min	13,000 rpm 20 min (Amicogen)	28.8 U	1,000	500
6	50 μL	150 μL	300 μL	15 min	13,000 rpm 20 min (Amicogen)	43.2 U	1,000	500

Smaple 1-2 : 1000 ppm extract

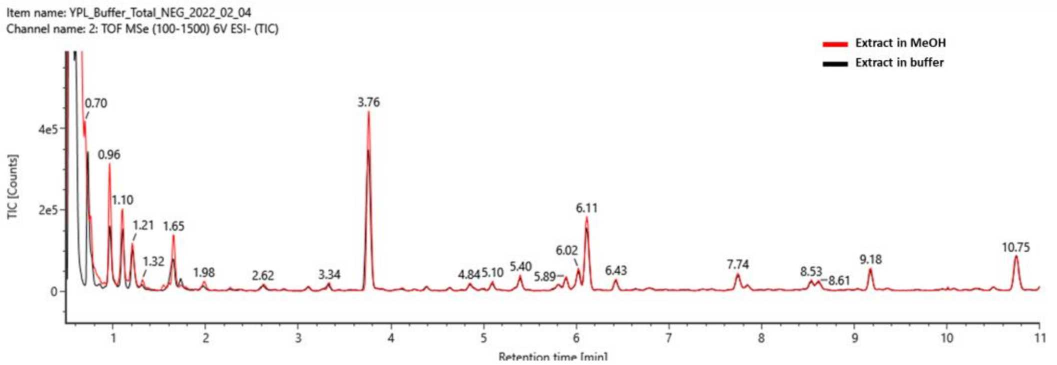

Smaple 3 : 1000 ppm extract + tyrosinase enzyme (14.4U) / 37˚C, 20 min / amicogen filter

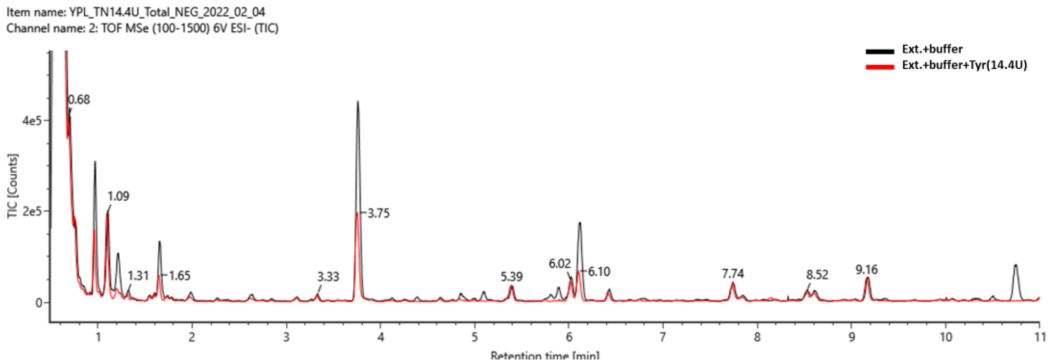

Smaple 4-6 : 1000 ppm extract + tyrosinase enzyme (14.4, 28.8, 43.2 U) / 37˚C, 15 min / amicogen filter

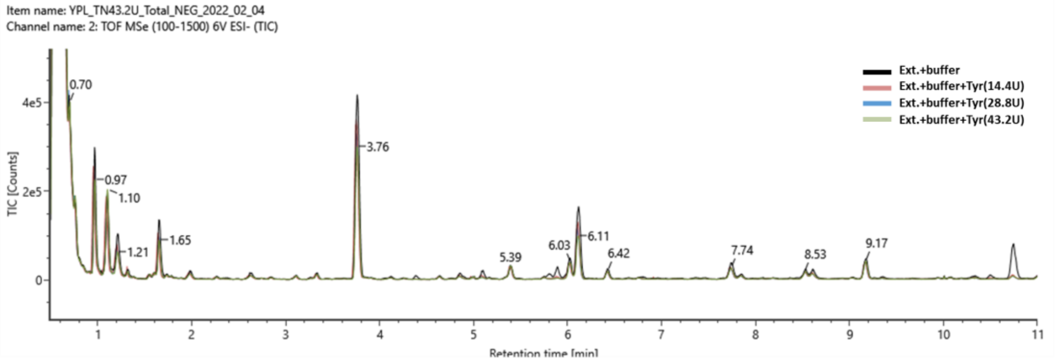

Table S2. Hydrogen bonding pattern of Verproside and Kojic acid on mushroom (mTyr) and human tyrosinases (hTyr). The donor and acceptor atoms are named and the distance between two assigned atoms are measured in unit of Å.

mTyr-Verproside

#	Donor		Acceptor		Dist
1	Ver	O39	HIS61	ND1	3.45
2	Ver	O39	HIS61	NE2	3.10
3	Ver	O41	HIS85	NE2	3.15
4	Ver	O20	VAL283	N	2.85
5	Ver	O28	HIS244	NE2	3.27
6	Ver	O41	HIS61	NE2	3.72
7	Ver	O41	HIS259	NE2	3.82

hTyr-Verproside

#	Donor		Acceptor		Dist
1	Ver	O22	ARG196	O	3.09
2	GLN378	NE2	Ver	O11	3.42
3	GLN378	NE2	Ver	O18	2.99
4	GLN378	NE2	Ver	O20	2.99

mTyr-Kojic_acid

#	Donor		Acceptor		Dist
1	Kojic	O11	GLY281	O	3.07
2	Kojic	O9	HIS296	NE2	3.56

hTyr-Kojic_acid

#	Donor		Acceptor		Dist
1	Kojic	O11	MET374	O	3.00
